# Low-intensity pulsed ultrasound/nanomechanical force generators enhance osteogenesis of BMSCs through microfilaments and TRPM7

**DOI:** 10.1186/s12951-022-01587-3

**Published:** 2022-08-13

**Authors:** Huan Yao, Liang Zhang, Shujin Yan, Yiman He, Hui Zhu, Yasha Li, Dong Wang, Ke Yang

**Affiliations:** 1 Pediatric Research Institute, Children’s Hospital of Chongqing Medical University, National Clinical Research Center for Child Health and Disorders, Ministry of Education Key Laboratory of Child Development and Disorders, China International Science and Technology Cooperation Base of Child Development and Critical Disorders, Chongqing Engineering Research Center of Stem Cell Therapy, Chongqing, 400014 China; 2 Department of Ultrasound, The First Affiliated Hospital of Chongqing Medical University, Chongqing, 400016 China

**Keywords:** Low-intensity pulsed ultrasound, Nanomechanical force generators, Mechanotransduction, Osteogenesis, Transient receptor potential melastatin 7

## Abstract

**Background:**

Low-intensity pulsed ultrasound (LIPUS) has been reported to accelerate fracture healing, but the mechanism is unclear and its efficacy needs to be further optimized. Ultrasound in combination with functionalized microbubbles has been shown to induce local shear forces and controllable mechanical stress in cells, amplifying the mechanical effects of LIPUS. Nanoscale lipid bubbles (nanobubbles) have high stability and good biosafety. However, the effect of LIPUS combined with functionalized nanobubbles on osteogenesis has rarely been studied.

**Results:**

In this study, we report cyclic arginine-glycine-aspartic acid-modified nanobubbles (cRGD-NBs), with a particle size of ~ 500 nm, able to actively target bone marrow mesenchymal stem cells (BMSCs) via integrin receptors. cRGD-NBs can act as nanomechanical force generators on the cell membrane, and further enhance the BMSCs osteogenesis and bone formation promoted by LIPUS. The polymerization of actin microfilaments and the mechanosensitive transient receptor potential melastatin 7 (TRPM7) ion channel play important roles in BMSCs osteogenesis promoted by LIPUS/cRGD-NBs. Moreover, the mutual regulation of TRPM7 and actin microfilaments promote the effect of LIPUS/cRGD-NBs. The extracellular Ca^2 +^ influx, controlled partly by TRPM7, could participated in the effect of LIPUS/cRGD-NBs on BMSCs.

**Conclusions:**

The nanomechanical force generators cRGD-NBs could promote osteogenesis of BMSCs and bone formation induced by LIPUS, through regulation TRPM7, actin cytoskeleton, and intracellular calcium oscillations. This study provides new directions for optimizing the efficacy of LIPUS for fracture healing, and a theoretical basis for the further application and development of LIPUS in clinical practice.

**Supplementary Information:**

The online version contains supplementary material available at 10.1186/s12951-022-01587-3.

## Introduction

The increasing older population results in a rising incidence of fractures, with approximately 16 million fractures occurring in the United States each year. When some risk factors, such as osteoporosis, diabetes, and smoking, are present, the fracture healing process can be delayed [1, 2]. Autografts are still the gold standard for bone defect repair, but its drawbacks such as the limited bone quantities, donor-site morbidity, and prolonged bed-rest drive the development of non-invasive treatment [3]. Allografts have the advantages of obtaining osteoconductivity and mechanical strength immediately, but the osteogenic and remodeling abilities is limited. Tissue engineering has been shown to be effective, but there are also deficiencies, such as abnormal bone formation, rapid bone degradation and cancer [4]. Low-intensity pulsed ultrasound (LIPUS), as a non-invasive mechanical stimulation, provides a convenient, tolerable and targeted treatment. It was officially approved in the United States to accelerate fresh fracture healing, and treat delayed union or nonhealing of bone fractures.

Bone marrow mesenchymal stem cells (BMSCs) are a subset of pluripotent stem cells with high proliferative activity, and the potential to differentiation into the osteoblast lineage is essential for bone formation [5, 6]. In a mouse fracture model, BMSCs were found to be present in the area of new bone formation [7]. The intracellular and extracellular mechanical microenvironment, and the signals generated by the mechanical strain play an important role in the regulation of MSCs behavior [8]. As a mechanical stress, LIPUS has the potential to promote the osteogenic differentiation of BMSCs and bone regeneration based on BMSCs [9, 10]. However, some studies suggest that the LIPUS treatment alone cannot be applied to treat all nonunion types. Due to the insufficient penetration and attenuation of ultrasound in soft tissue, LIPUS is not effective enough in the treatment of deep fractures such as hip fractures [11, 12]. Long-term treatment with LIPUS results in limited patient compliance [13]. Therefore, further research is urgently needed to optimize the therapeutic effect of LIPUS to reduce the duration of the corresponding treatment and expand therapeutic range.

The mechanical properties of cells depend to a large extent on the structure of the cytoskeleton, which is composed of microfilaments, microtubules, and intermediate filaments [14]. Actin is the main component of eukaryotic cell microfilaments. The microfilaments in the cytoplasm have two forms of exist: polymerized fibrous (F) actin and monomeric globular(G) actin [15]. The dynamic changes between F-actin and G-actin play a key role in cell response to external mechanical stress [16]. Integrin is connected to the actin microfilament, and can transmit extracellular mechanical signals into cells through actin microfilament [17]. The continuous dynamic changes of actin regulate cell proliferation and differentiation, and mediate microenvironment sensing and intracellular signal transduction [18]. Transient receptor potential melastatin 7 (TRPM7) is a mechanically sensitive plasma membrane calcium channel, which participates in the regulation of multiple physiological processes in cells [19]. Previous studies have shown that TRPM7 defects can hinder bone formation [20]. The promotion of fracture healing by LIPUS can be achieved by BMSC sensing the mechanical stimulation of LIPUS through specific mechanisms.

Gas filled microbubbles can amplify the sound pressure and enhance the biological effects of ultrasound, improving the therapeutic effect of LIPUS [21–23]. The ultrasonic pulse produces volume pulsation of the microbubbles and generate fluid microflow around the microbubbles, resulting in local shear force on the cells [24]. Previous studies have shown that lipid microbubbles coated with arginine–glycine–aspartic acid (RGD) peptides can target the integrin receptors on the cell membrane and attach to the cell through the RGD peptide–integrin binding. Furthermore, the directional ultrasound radiation force causes the microbubbles attached to the cell surface to undergo translational motion without separating from the cells, which generates subcellular strain and stress through the microbubble–integrin–cytoskeleton connection. During the interval between ultrasound pulses, the microbubbles return to their original position on the cell membrane. Therefore, LIPUS combined with targeted microbubbles can induce the controllable cyclic subcellular mechanical stress on BMSCs, which can recover osteogenic differentiation inhibited on the soft matrix [24, 25].

Ordinary lipid microbubbles still have some limitations in practical applications, due to their large size, low stability, and poor vascular penetration ability [26]. With the rapid development of nanotechnology, nanoscale lipid bubbles (nanobubbles) encapsulated in phospholipid shells exhibit high stability, good biosafety, and vascular penetration ability [27, 28]. Therefore, nanobubbles may represent a more effective option in ultrasound application. Nanobubbles conjugated with cyclic RGD could target and bind integrin receptors on cell membranes and improve the mechanical effect of LIPUS. However, studies exploring the combination of LIPUS and cyclic RGD-modified nanobubbles (cRGD-NBs) in BMSCs osteogenesis are relatively scarce.

In summary, in this study we aimed to design and prepare nanobubbles that can actively target BMSCs, with the purpose to determine whether the combination with targeted nanobubbles can enhance the effect of LIPUS in promoting the osteogenesis of BMSCs and bone formation. In addition, we sought to understand the possible molecular mechanisms involved in this process. The results may provide new directions for optimizing the efficacy of LIPUS in promoting fracture healing in clinical practice.

## Materials and methods

### Preparation and characterization of cRGD-NBs

The nanobubbles were prepared as described previously [29]. Briefly, 2 mg 1,2-distearoyl-sn-glycero-3-phosphoethanolamine-[(polyethylene glycol)-2000]-cRGD (DSPE-PEG2000-cRGD) was mixed with 5 mg dipalmitoylphosphatidylcholine (DPPC, Ruixi Biological Technology, Xi’an, China) and phosphate-buffered solution (PBS) with 10% glycerin. The mixture was incubated in a 50 °C water bath for 30 min and then cooled down to room temperature with slow and continuous stirring. After C_3_F_8_ gas injection, the mixture was mechanically vibrated using an amalgam capsule mixer bender (Modal YJT-2, Shanghai Medical Equipment Co., Ltd., Shanghai, China) at 4000 rpm for 90 s. After low-speed centrifugation (300 rpm, 5 min) repeated twice, high-speed centrifugation (1000 rpm, 5 min) was used to purify cRGD-NBs. The ordinary nanobubbles (NBs) were prepared using 2 mg 1,2-distearoyl-sn-glycero-3-phosphoethanolamine-[(polyethylene glycol)-2000]-Maleimide (DSPE-PEG2000-MAL, Ruixi Biological Technology), following the same method. The purified nanobubbles were stored at 4 °C for further experiments. Commercial sulfur hexafluoride microbubbles (MBs, SonoVue, Bracco, Italy) were used in this study. 5 mL of sterile saline was added to the vial prior to use and shaken vigorously to form a homogeneous white emulsion. The concentrations of the above materials were measured by a hemocytometer.

The particle size and zeta potential data were determined using a Zetasizer Nano ZS instrument (Malvern Instruments, Malvern, UK). The morphology, size, and distribution of cRGD-NBs and NBs were observed under an inverted microscopy (Nikon Eclipse Ti, Tokyo, Japan). Transmission electron microscopy (TEM, Hitachi H-7600, Tokyo, Japan) was used to observe the morphology and structure of the samples in detail. X-ray photoelectron spectroscopy (XPS) was performed on a Thermo Scientific K-Alpha spectrometer to analyze the spectra of cRGD-NBs and NBs.

### Determination of cRGD-NBs targeting ability and cytotoxicity

Mouse BMSCs were isolated from 5-week C57BL/6 J mice, as described previously [30, 31]. The cells were seeded on a micro cell culture dish for laser scanning confocal microscopy (C2 Plus, Nikon, Tokyo, Japan) and maintained in α-modified Eagle’s Medium (α-MEM, HyClone, Logan, UT) with 10% fetal bovine serum (FBS, Gibco) for 24 h. The cells were divided into NBs and cRGD-NBs groups and treated with fresh medium. Then, 100 μL of nanobubbles labeled with 1,1′-dioctadecyl-3,3,3′,3′-tetramethylindocarbocyanine perchlorate (Dil, Beyotime, Shanghai, China) was added to each group. After 90 min of culturing, the cells were fixed with 4% paraformaldehyde (Leagene, Beijing, China), washed with PBS one time, and stained with 2-(4-amidinophenyl)-6-indolecarbamidine dihydrochloride (DAPI) (1:150, Leagene) for 5 min. After washing with PBS for three times, the cells were observed under a laser scanning confocal microscopy. For the cytotoxicity assay, cells were placed in a 96-well plate and divided in four groups: control, MBs, NBs, and cRGD-NBs. The cells were treated with 10 μL each of PBS (control), MBs, NBs, and cRGD-NBs for 90 min per day, and the medium was changed. Sixty minutes before the end of the culture on day 1, 2, or 3, 110 μL of 9% CCK8 (Dojindo, Kumamoto, Japan) was added to each well. The absorption of solubilized formazan was measured at the wavelength of 450 nm using a microplate reader (Synergy HT, BioTek, Winooski, VT).

### LIPUS treatment

BMSCs at 3–6 passages were grown in α-MEM with 10% FBS and then added to an osteogenic differentiation medium consisting of α-MEM + 10% FBS + 0.1 μmol/L dexamethasone (Solarbio, Beijing, China) + 10 mmol/L β-glycerophosphate (Solarbio) + 50 μg/mL ascorbic acid (Solarbio). The medium was changed every 2–3 days. The cells were treated with LIPUS (2776, Chattanooga, TN) at a frequency of 3 MHz, an intensity of 100 mW/cm^2^, and a duty cycle of 50%. The quality of ultrasonic waveforms at different intensities was monitored using a digital oscilloscope (Tektronix MSO58, Beaverton, OR). cRGD-NBs were mixed with the medium daily, cultured at 37 °C for 90 min, and treated with LIPUS for 10 min, followed by refreshing the medium (when cells were plated onto 12- or 24-well dishes, we added 150 or 60 μL of nanobubbles per well). The cytoskeletal interference procedure was as follows: 2 h after the combined LIPUS and cRGD-NBs treatment, the cells were treated with 0.2 μg/mL cytochalasin D (CytoD, Rhawn, Shanghai, China) or 25 nmol/L jasplakinolide (JA, R&D Systems, Minneapolis, MN) for 2 h, and the medium was refreshed. The working concentration of DMSO was 0.1% (v/v).

### Live/dead assay

BMSCs were treated with PBS (control), LIPUS, LIPUS + NBs, and LIPUS + cRGD-NBs for 3 days. After washing with PBS three times, the cells were stained with calcein AM and propidium iodide (Beyotime) for viable and dead cells, according to the manufacturer’s instructions. The cells were then observed under a laser scanning confocal microscopy.

### Cell apoptosis assay

The cells were treated for 3 days and then harvested by trypsinization (Beyotime). After washing with PBS three times, 1 × 10^6^ cells were resuspended in 500 μL PBS. Apoptosis was assayed by fluorescence-activated cell sorting (FACS, CytoFLEX Flow Cytometer, Becton Coulter, Brea, CA) after annexin V and PI staining.

### Alkaline phosphatase (ALP) staining

The cells were seeded into a 24-well plate and treated for 7 days. The ALP working solution was prepared as follows: the naphthol AS-MX phosphate alkaline solution/double-distilled water (ddH_2_O) volume ratio was set to 1:25, and FAST-BLUE (Sigma-Aldrich, St. Louis, MO) was added until the solution turned light yellow. The volume of working solution per well was 250 µL. After washing with PBS, the cells were fixed with 4% paraformaldehyde for 1 min, followed by washing once with PBS. The cells were stained with the ALP working solution at 37 °C for 30 min. When the yellow dye was metabolized to blue deposits, ALP staining was observed under a microscope and quantified using the ImageJ 1.4.3.67 (NIH, Bethesda) and GraphPad Prism 7.0 (GraphPad Inc, San Diego, CA) software packages.

### Alizarin red s staining

At day 21, the culture plates were placed for 1 min in 4% paraformaldehyde, and subsequently stained for 30 min using Alizarin Red S solution (iCell Bioscience Inc, Shanghai, China). Then, the cells were washed with PBS, and calcium nodule formation was captured under a microscope. To quantify calcium deposition, the cells were destained using 10% cetylpyridinium chloride (Macklin, Shanghai, China), after which the extracted dye was transferred to a 96-well plate, and the optical density was measured at 405 nm.

### Western blot (WB) analysis

The cells were seeded in a 12-well plate and treated for 14 days. Aliquots of cell lysates containing 10 μg of proteins were separated by 12% sodium dodecyl sulfate (SDS)–polyacrylamide gel and transferred to a polyvinylidene fluoride (PVDF) membrane (EMD Millipore, Billerica, MA). The membranes were blocked with blocking solution (Beyotime) for 15 min and then incubated with anti-runt-related transcription factor 2 (RUNX2) (rabbit monoclonal antibody, 1:500, Abcam, Cambridge, UK), anti-osteopontin (OPN) (rabbit polyclonal antibody, 1:1000, Sigma-Aldrich), anti-osteocalcin (OCN) (rabbit polyclonal antibody, 1:500, Abcam), anti-collagen I (COLI) (rabbit polyclonal antibody, 1:500, Wanleibio, Shenyang, China), anti-TRPM7 (rabbit polyclonal antibody, 1:1000, Abcam), and anti-glyceraldehyde-3-phosphate dehydrogenase (GAPDH) (mouse monoclonal antibody, 1:1000, Abcam). This was followed by the addition of goat anti-rabbit IgG secondary antibody (1:4000, Biosharp, Guangzhou, China), rabbit anti-mouse IgG-HRP secondary antibody (1:10,000, ZEN BIO, Chengdu, China), and ECL visualization of the bands (Bio-Rad, Hercules, CA). The intensity of each WB band was analyzed using the ImageJ 1.4.3.67 and GraphPad Prism 7.0 software packages.

### In vivo bone regeneration

The animal experiment procedure was strictly conducted in compliance with Ethics Committee of the First Affiliated Hospital of Chongqing Medical University. Male C57BL/6 J mice of 5–6 weeks of age were purchased from Laboratory Animal Center of Chongqing Medical University. After the mouse was anesthetized with pentobarbital sodium, 3-mm diameter calvarial defect was created by continuous irrigation on the parietal bone with a trephine drill. Following the establishment of the calvarial defect models, mice were randomly divided into four groups (*n* = 6). Then, mice were locally injected with 100 µL of saline mixed with cRGD-NBs or NBs (1 × 10^9^ bubbles/mL) at the defect regions as LIPUS + cRGD-NBs group or LIPUS + NBs group, and the control and LIPUS groups were injected with 100 µL of saline. 90 min later, the mice were anesthetized with pentobarbital sodium and treated with or without LIPUS for 10 min following the same parameters as above. The above processes were carried out once a day. Mice in all groups were sacrificed after 4 weeks-treatment and the calvarium of each mouse were harvested for micro-CT (Always Imaging, Shanghai, China) investigation.

### F-actin/G-actin assay

F-actin and G-actin contents in a cell population were analyzed using a F-actin/G-actin assay kit, according to the manufacturer’s instructions (Cytoskeleton, Denver, CO). Briefly, the cells were treated for 3 days and lysed with prewarmed LAS2 lysis buffer. The cell lysate was centrifuged for 5 min at 350 × *g* to remove the cell debris, followed by ultracentrifugation at 20,000 × *g* for 1 h at 4 °C. The F-actin in the pellet was resuspended and incubated with 100 μL F-actin depolymerization buffer. Equal volumes of G-actin in the supernatant and F-actin fractions were mixed with 5 × SDS sample buffer and run on SDS–polyacrylamide gel electrophoresis. WB analysis was performed using rabbit anti-actin polyclonal antibody (1:500) and the intensity of each WB band was analyzed using ImageJ 1.4.3.67.

### Immunostaining assay

Immunofluorescence staining was performed as described in previous reports [32, 33]. Briefly, the cells were grown on coverslips and treated for 3 days. After washing with PBS, the cells were fixed with 4% paraformaldehyde, permeabilized with PBS containing 0.1% (v/v) Triton X-100 (Solarbio), and incubated with 5% bovine serum albumin (Solarbio). After that, the cells were incubated with anti-TRPM7 (rabbit polyclonal antibody, 1:80, GeneTex, Irvine, CA) or anti-RUNX2 (rabbit monoclonal antibody, 1:500, Abcam) primary antibody at 4 °C overnight, followed by incubating with secondary antibody at room temperature for 1 h. The secondary antibody was DyLight 549 goat anti-rabbit IgG (1:200, Abbkine, Wuhan, China) or CoraLite488-conjugated goat anti-rabbit IgG (1:100, Proteintech, Rosemont, IL). If the cells needed microfilaments staining, they were incubated with Actin-Tracker Green (Beyotime) according to the manufacturer’s instructions. The cells were then counterstained with DAPI for 5 min and mounted with an anti-fluorescence quenching agent (Solarbio). The stained cells were observed under a laser scanning confocal microscope. The ImageJ 1.4.3.67, GraphPad Prism 7.0, and Origin 2019 (OriginLab Corporation, Northampton, MA) software packages were used for image processing.

### TRPM7 protein levels in membrane and cytosol

The membrane and cytosol proteins were extracted from 1 × 10^7^ cultured cells using a commercial kit (KeyGEN, Nanjing, China), according to the kit manual. Membrane proteins were concentrated by treatment with trichloroacetic acid and acetone, mixed with loading buffer, and denatured by boiling. The TRPM7 levels in the membrane and cytosol proteins were detected by WB, as described above. Na^+^-K^+^ ATPase and GAPDH were used as internal controls for membrane and cytosol proteins, respectively.

### Calcium imaging

The cells were incubated with 2.5 μmol/L Fluo-4 AM (Beyotime) at 37 °C for 10 min. After washing with PBS, Opti-α-MEM was added and cells were observed under a laser scanning confocal microscope. The total observation and baseline times were 12 min and 30 s, respectively, whereas the LIPUS exposure period was 0.5–2.5 min; the treatment parameters were the same as those described in the “LIPUS treatment” subsection, and the interval between image captures was 4 s. The calcium response of each frame of image was quantified as Δ*F*/*F*_0_, where *F*_0_ is the average baseline fluorescence intensity before LIPUS processing, Δ*F* = *F*_P_ − *F*_0_, and *F*_P_ is the real-time fluorescence intensity [34]. We compared the highest *F*_P_ values of each group during and after LIPUS treatment. The analysis was carried out using the NIS-Elements AR 4.20 (Nikon), GraphPad Prism 7.0, and Origin 2019 software packages.

### Small interfering RNA (siRNA) interference

The cells were seeded on 24-well plates and divided into control, negative control (NC, non-targeting siRNA control, 5′-UUCUCCGAACGUGUCACGUTT-3′), siRNA#1 (5′-GGGAGUGUGUAUAUAUUAUTT-3′), siRNA#2 (5′-GAGCCCAACAGAUGCUUAUTT-3′), and siRNA#3 (5′-GGGCAUCUCUAUAUCAUUATT-3′) groups to identify the best siRNA sequence that interfered with TRPM7. This sequence was then used for the subsequent experiments. Two microliters of Lipofectamine 2000 (Invitrogen, Carlsbad, CA) was mixed with 50 μL Opti-α-MEM and left standing at room temperature for 5 min (labeled as reagent 1). A 2.5 μL aliquot of siRNA (GenePharma, Shanghai, China) was mixed with 50 μL Opti-α-MEM and left standing at room temperature for 5 min (labeled as reagent 2). Reagent 1 was dropped onto reagent 2, gently mixed and left standing at room temperature for 20 min, and then transfected immediately. During the standing period, 400 μL of fresh Opti-α-MEM medium was added to each well, followed by the addition of 100 μL of the transfection mixture and gentle shaking. After 4–6 h incubation at 37 °C, the medium was replaced with fresh α-MEM medium containing FBS and the TRPM7 protein levels were detected after 2 days.

### Statistical analysis

All experiments were performed at least three times. Data are presented as mean ± standard deviation (SD). Student’s *t*-test was used to assess the differences between two groups, whereas ANOVA was used to evaluate the differences between three or more groups. A *p* value of less than 0.05 was considered statistically significant. The GraphPad Prism 7.0 software was used for statistical analysis.

## Results

### Characterization of cRGD-NBs

**Scheme 1:**
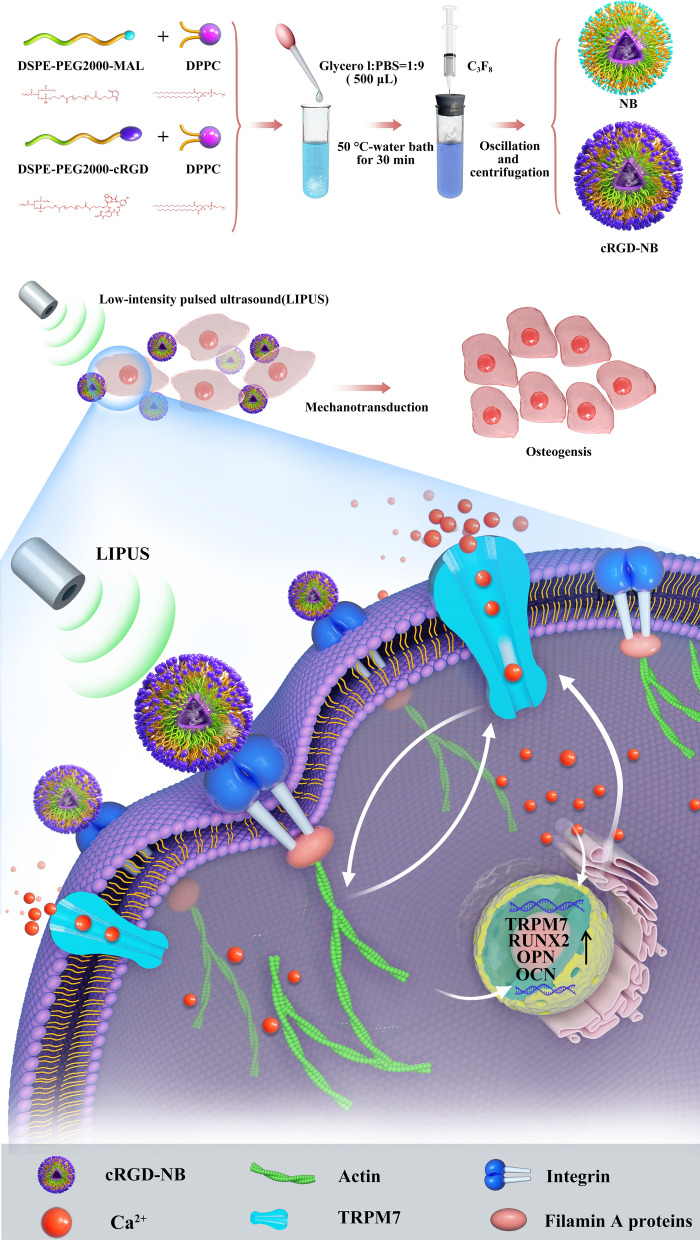
Schematic illustrations of (top) synthesis process of cRGD-NBs and NBs and (bottom) molecular mechanism behind enhanced osteogenic differentiation of the LIPUS/cRGD-NBs combination on BMSCs

**Fig. 1:**
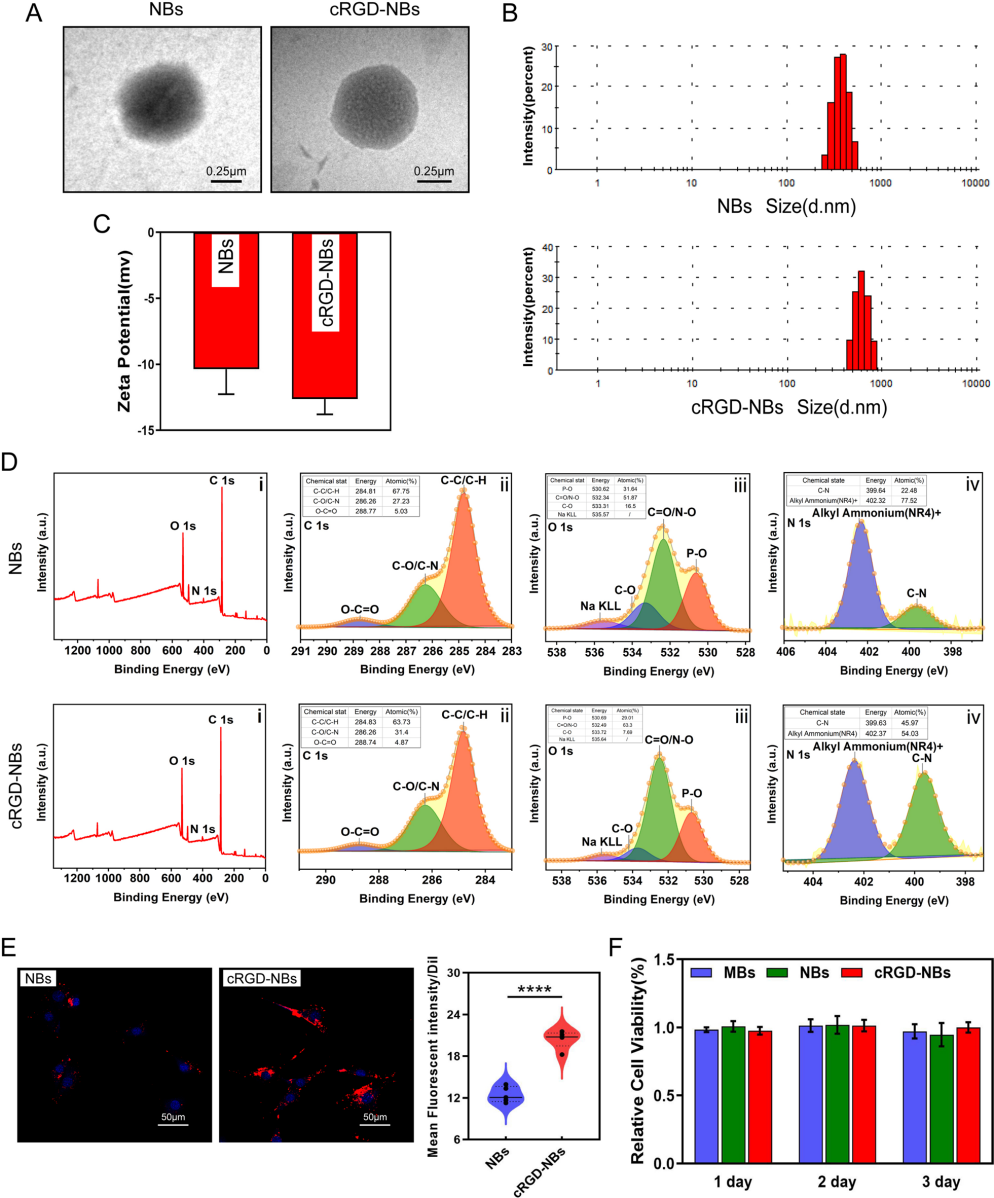
Characterization of cRGD-NBs and NBs. **A** TEM images of NBs and cRGD-NBs 6 h after preparation. **B** Particle size distributions of NBs and cRGD-NBs. **C** Zeta potentials of NBs and cRGD-NBs. **D** XPS profiles of NBs and cRGD-NBs: (i) full survey and high-resolution (ii) C 1s, (iii) O 1s, and (iv) N 1s spectra. **E** Representative fluorescence images of Dil-labeled NBs and cRGD-NBs co-incubated with BMSCs and corresponding quantitative analysis. **F** CCK8 results of MBs, NBs, and cRGD-NBs groups after 1–3 days of culture. Data are presented as mean ± SD (*n* = 3). ****P < 0.0001

In this study, we used DSPE-PEG2000-cRGD and DSPE-PEG2000-MAL mixed with DPPC to prepare cRGD-NBs and NBs, respectively (Scheme 1). Light microscopy observations of cRGD-NBs, NBs, and MBs at 0 h after preparation revealed a regular morphology, good dispersion, and a large number of bubbles. After 6 h, the number of MBs decreased, while the number of cRGD-NBs and NBs did not change significantly. Moreover, only a small amount of cRGD-NBs was observed after 24 h (Additional file 1: Fig. S1). TEM images showed that cRGD-NBs and NBs appeared as round vesicles (Fig. 1A). Dynamic light scattering (DLS) analysis revealed that the average diameters of cRGD-NBs and NBs were 577.5 ± 102.2 and 441.8 ± 69.44 nm, respectively (Fig. 1B), whereas their zeta potentials were − 12.6 ± 0.9 and − 10.4 ± 1.5 mV, respectively (Fig. 1C). The concentration of cRGD-NBs and NBs was 1 × 10^9^ bubbles/mL, and the concentration of MBs was 1 × 10^8^ bubbles/mL. XPS was used to compare the chemical bonds of cRGD-NBs and NBs (Fig. 1D). The main differences were observed in the atomic percentages of certain functional groups, such as C–O/C–N, C = O/N–O, and C-N, as shown by the survey, C 1s, O 1s, and N 1s spectra. A significantly higher atomic percentage of functional groups such as C = O/N–O (O 1s spectra) and C–N (N 1s spectra) was found on the cRGD-NBs than NBs surface, which is consistent with the chemical structure of the raw materials used to prepare them.

To evaluate the ability to target cells, BMSCs were incubated with Dil-labeled NBs and cRGD-NBs, and the fluorescent signals were observed under a confocal microscopy. Compared with NBs, more red fluorescence was observed on the cell surface incubated with cRGD-NBs (Fig. 1E), which indicates that cRGD-NBs had a better ability to cell targeting. To examine the cytotoxic effects of cRGD-NBs, NBs, and MBs, the cytoactive of BMSCs was assessed by CCK8 assay. As shown in Fig. 1F, no change in cell viability was observed after incubating with cRGD-NBs, NBs, or MBs for 1, 2, and 3 days.

### Condition optimization and biosafety evaluation of LIPUS/cRGD-NBs combination

The temporal waveforms of LIPUS at 100 mW/cm^2^, 200 mW/cm^2^, and 300 mW/cm^2^ are illustrated in Additional file 1: Fig. S2. In order to eliminate the temperature influence of LIPUS, the thermal images of BMSCs were acquired using an infrared thermography camera. After LIPUS was exposed to 100 mW/cm^2^ with or without cRGD-NBs for 10 min, the cells had temperature rise of less than 1 °C. However, the ΔT values were more than 2 °C and 4.5 °C at the intensity of 200 mW/cm^2^ and 300 mW/cm^2^, respectively (Additional file 1: Fig. S3–5). Next, the ALP activity for osteogenesis was assessed and treated with 100 mW/cm^2^ LIPUS and cRGD-NBs on day 7 and day 14. LIPUS treatment for 5 min, 10 min and 15 min per day could increase the activity of ALP, and the highest ALP activity was obtained in 10 min of treatment (Additional file 1: Fig. S6).

**Fig. 2:**
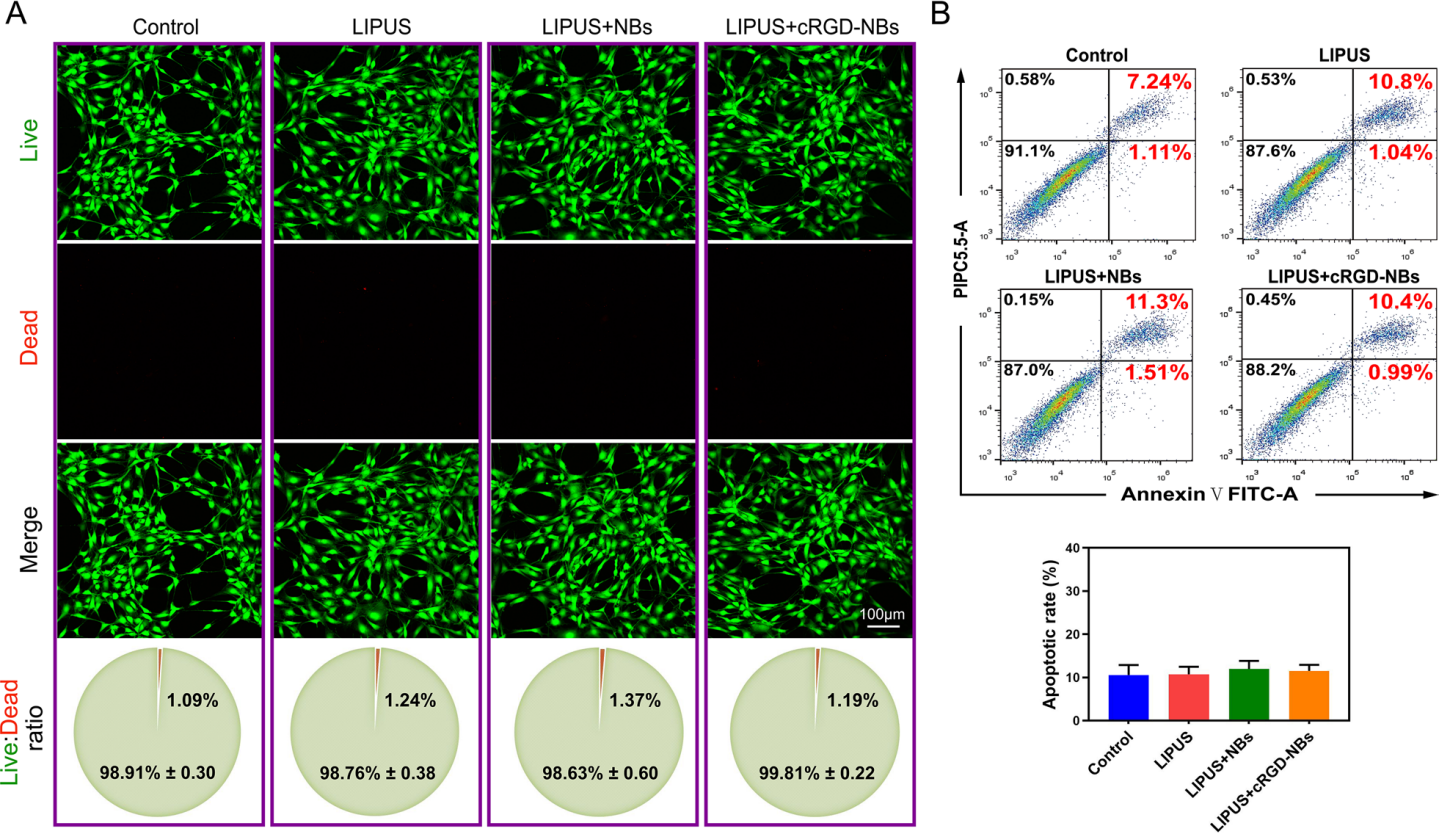
Biosafety evaluation of LIPUS/cRGD-NBs combination. **A** Results of Live (green)/Dead (red) fluorescence assay of control, LIPUS, LIPUS + NBs, and LIPUS + cRGD-NBs groups after 3 days of treatment. **B** Flow cytometric detection of cell apoptosis after 3 days of treatment in each group

We also evaluated the effect of combining LIPUS with cRGD-NBs or NBs on the cell viability through a Live/Dead assay. The results show that, after 3 days of culture, the cells in each group remained viable, with a ~ 1% proportion of dead cells and no statistical difference between the groups (Fig. 2A). Consistent with these findings, no statistical difference was observed between the rates of cell apoptosis or the percentages of cell proliferation of different groups measured by flow cytometry (Fig. 2B, Additional file 1: Fig. S7). These results show that the combination of LIPUS with cRGD-NBs had good biocompatibility, which provides the basis for the experiments described in the following section.

### Enhanced osteogenic activity and bone formation of LIPUS combined with cRGD-NBs

**Fig. 3:**
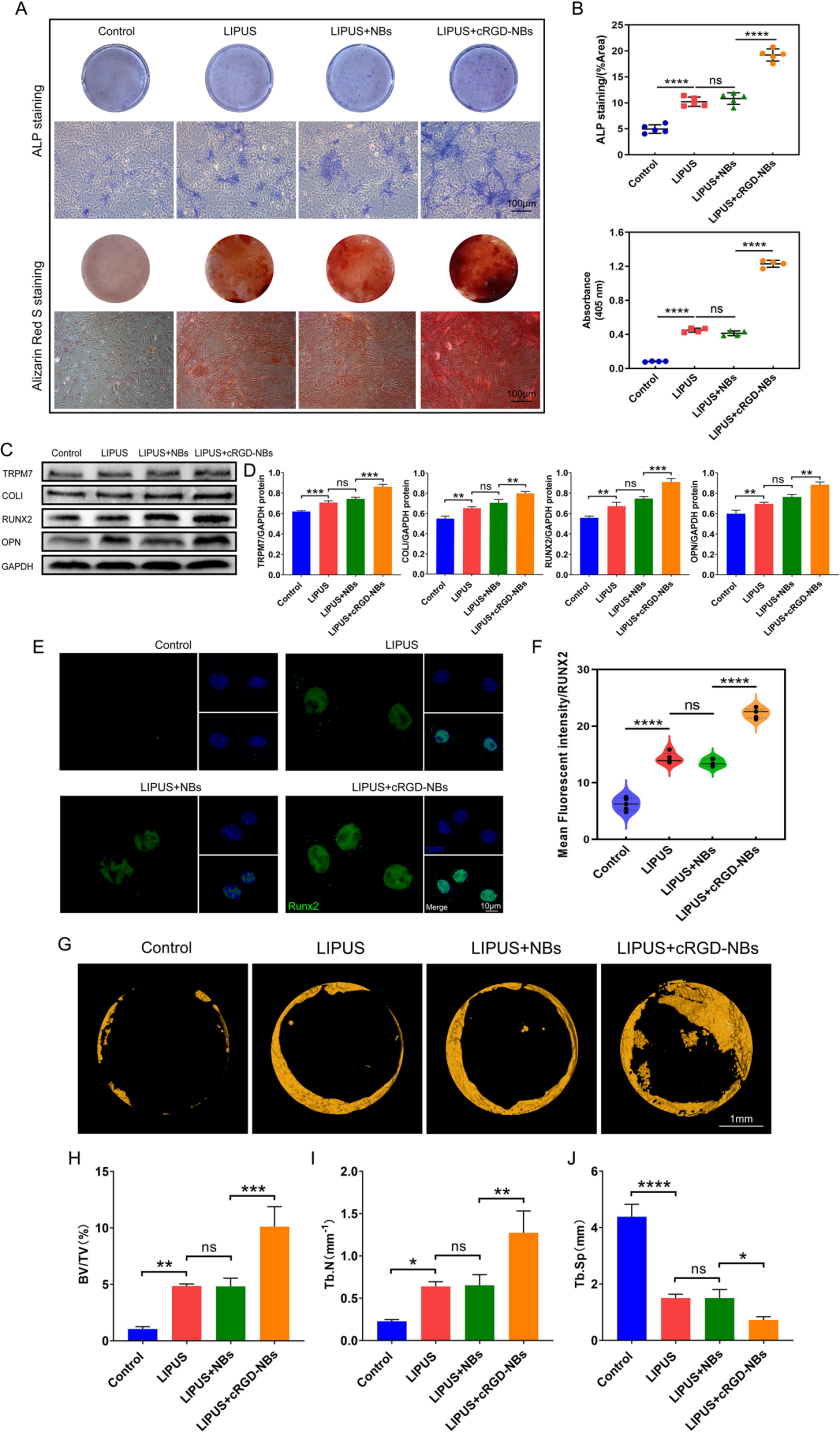
**A** ALP staining and Alizarin Red S staining on day 7 and 21, respectively: macroscopic (top) and microscopic (bottom) images. **B** Quantitative assessment of staining indicators in (**A**). **C** WB analysis of the effect of each group on the level of osteogenic differentiation-related RUNX2, OPN, COLI, and TRPM7 proteins after 14 days of treatment. **D** Quantification of immunoreactive bands observed in WB analysis. **E** Immunofluorescence images stained with RUNX2 (green) and DAPI (blue). **F** Quantitative analysis of RUNX2 fluorescence intensity. **G** Micro-CT images of mouse calvarial bone regeneration. **H–J** Quantitative analysis of bone volume/total volume (BV/TV), trabecular number (Tb.N), and trabecular spacing (Tb.Sp) calculated based on micro-CT analysis (*n* = 6 per group). Data are presented as mean ± SD (*n* = 3). *P < 0.05, **P < 0.01, ***P < 0.001, ****P < 0.0001

We next investigated whether the ability of LIPUS to promote osteogenesis can be further enhanced upon combining it with cRGD-NBs. ALP staining and Alizarin Red S staining were performed on day 7 and 21, respectively. The blue-purple staining and calcium nodule deposition in the LIPUS group were significantly increased compared with those of the control group, indicating that LIPUS upregulated the osteogenic differentiation ability of BMSCs. Compared with the LIPUS group, the LIPUS + NBs group did not show statistically significant differences in the ALP staining and Alizarin Red S staining, while the LIPUS + cRGD-NBs group showed significantly more positive staining areas than the LIPUS + NBs group (Fig. 3A, B). WB analysis was performed to analyze the levels of osteogenic differentiation-related RUNX2, OPN, and COLI proteins after 14 days of treatment in each group. The levels of these proteins were significantly higher in the LIPUS group than in the control group, but no significant difference was observed between the LIPUS + NBs and LIPUS groups. Moreover, the protein levels in the LIPUS + cRGD-NBs group were significantly higher than those in the LIPUS + NBs group. Interestingly, the protein level of the TRPM7 also showed the same trend of osteogenic differentiation-related proteins (Fig. 3C, D). Immunofluorescence staining showed that the expression of RUNX2 was mainly in the nucleus, and the quantitative analyses observed the trend in expression pattern was similar to that of the TRPM7 expression (Fig. 3E, F).

Next, a mouse calvarial defect model was constructed to explore the in vivo bone repair and regeneration ability of LIPUS combined with cRGD-NBs. Micro-CT analysis showed remarkable bone defect healing in the LIPUS group compared to the control group after 4 weeks of treatment, whereas there was no significant difference in bone healing in the LIPUS + NBs group compared with the LIPUS group. Notably, bone healing showed significant in LIPUS + cRGD-NBs group, compared to the LIPUS + NBs group (Fig. 3G, Additional file 1: Fig. S8). Further quantitative analysis revealed that the LIPUS + cRGD-NBs group had significantly higher bone volume/total volume (BV/TV) and trabecular number (Tb.N), as well as significantly lower trabecular spacing (Tb.Sp) than the LIPUS + NBs group (Fig. 3H–J). These results show that the combination of LIPUS with cRGD-NBs significantly improved the ability of LIPUS to promote osteogenesis and bone formation in vivo.

### Upregulation of mechanosensitive ion channel TRPM7 expression by LIPUS/cRGD-NBs combination

**Fig. 4:**
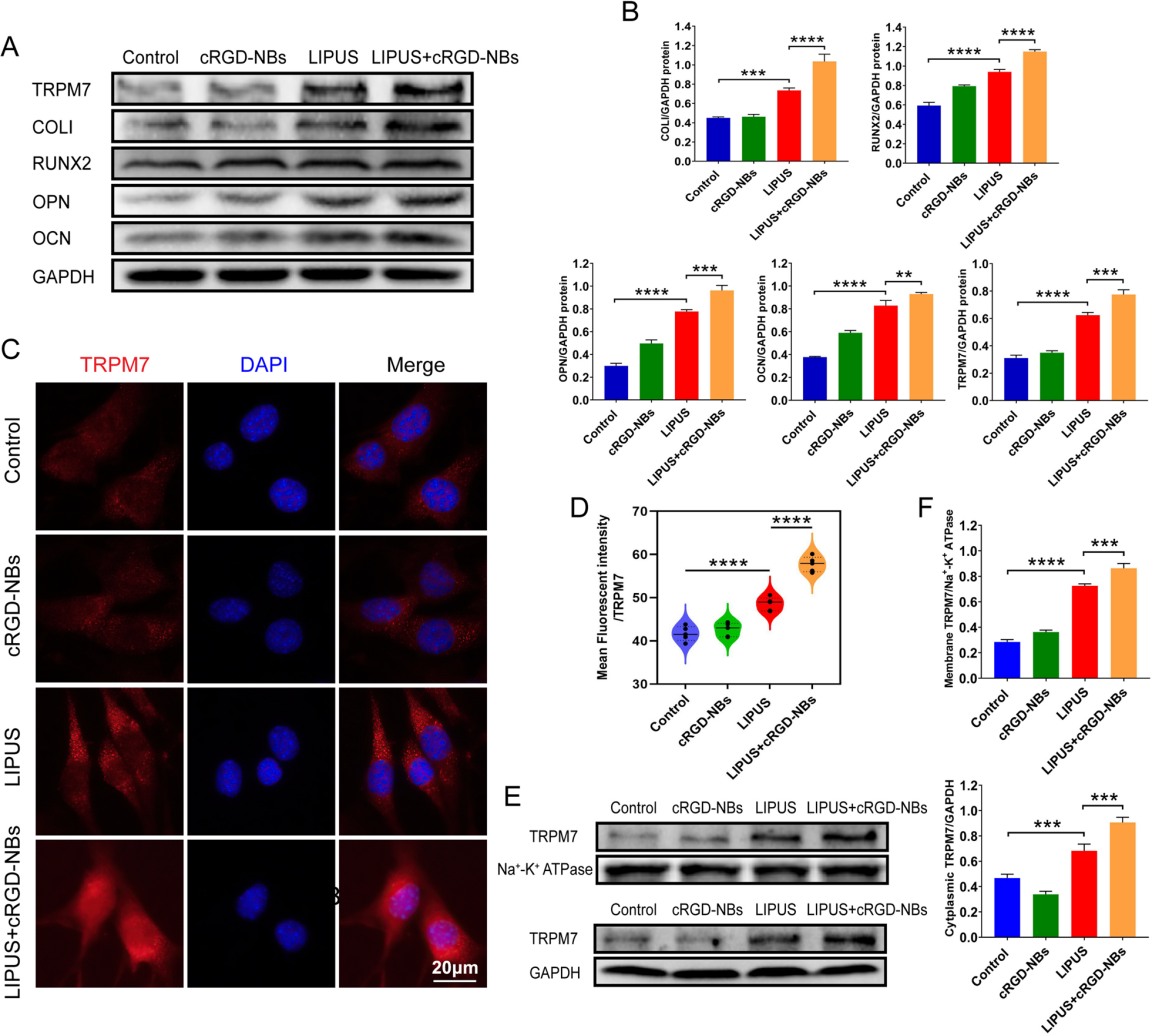
**A** WB analysis of the effect of each group on the level of osteogenic differentiation-related proteins (COLI, RUNX2, OPN, OCN, and TRPM7) after 14 days of treatment.** B** Quantification of immunoreactive bands in (**A**). **C** Immunofluorescence images stained with TRPM7 (red) and DAPI (blue) in each group. **D** Quantitative analysis of TRPM7 fluorescence intensity in panel C. **E** WB analysis of membrane and cytoplasmic TRPM7 levels in each group. **F** Quantification of immunoreactive bands in panel E. Data are presented as mean ± SD (*n* = 3). **P < 0.01, ***P < 0.001, ****P < 0.0001

The WB assay showed significantly increased protein levels of the COLI, RUNX2, OPN, and OCN in the LIPUS + cRGD-NBs group compared with the LIPUS group, while the treatment of cRGD-NBs alone did not increase the expression of COLI, RUNX2, OPN, and OCN compared with control group (Fig. 4A, B). Next, the expression of the mechanosensitive ion channel TRPM7 was assessed during this process. The protein level of TRPM7 had no difference in cRGD-NBs group compared with the control group, while the LIPUS + cRGD-NBs group showed significantly higher levels than the LIPUS group (Fig. 4A, B). The TRPM7 immunofluorescence analysis also showed a stronger fluorescence intensity in cytoplasm for the LIPUS + cRGD-NBs group than the LIPUS group (Fig. 4C, D). The WB analysis of TRPM7 membrane protein and cytoplasmic protein levels showed the same trend (Fig. 4E, F). These data indicated that the cRGD-NBs promoted osteogenesis effect of LIPUS and the expression of TRPM7 upregulated by LIPUS.

### Increased cytoskeletal actin polymerization by LIPUS/cRGD-NBs treatment

**Fig. 5:**
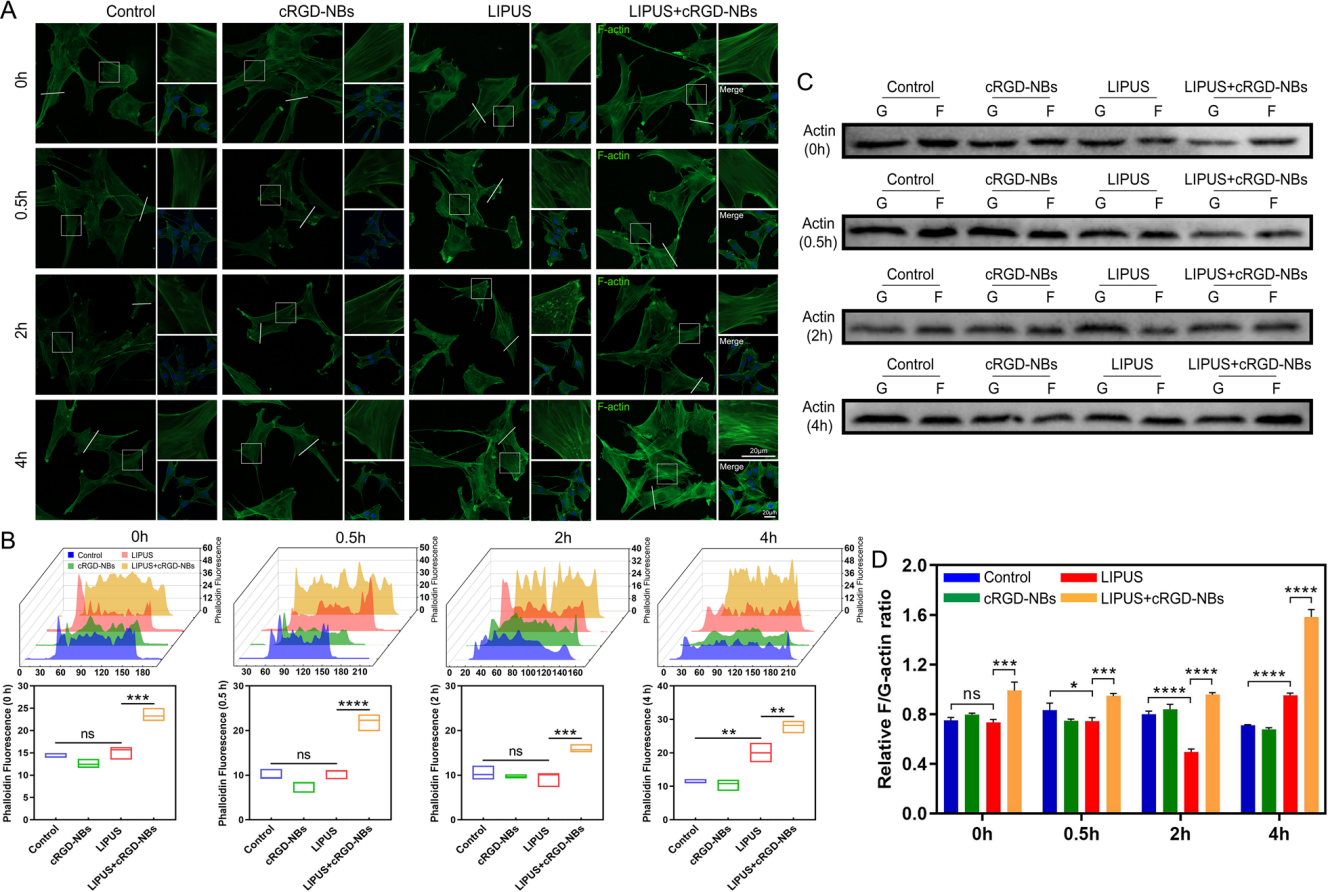
**A** Fluorescence images of each group after treatment for 0, 0.5, 2, and 4 h: microfilaments (green) and DAPI (blue). **B** Quantitative analysis of actin fluorescence intensity along the white lines in panel A. **C** WB analysis of F-actin and G-actin levels in each group after treatment for 0, 0.5, 2, and 4 h. **D** Quantification of immunoreactive bands in panel C. Data are presented as mean ± SD (*n* = 3). *P < 0.05, **P < 0.01, ***P < 0.001, ****P < 0.0001

The dynamic changes of actin microfilaments were performed to observe with fluorescent phalloidin at different time points after treatment in each group. At 0.5 h after LIPUS treatment, a few fluorescent spots were distributed in the cytoplasm, while at 2 h, the spots increased significantly and gathered around the nucleus, but the filamentous F-actin was significantly reduced. After 4 h, the fluorescence intensity of filamentous F-actin increased, and a small number of spots were still observed in the cells. However, after LIPUS treated with cRGD-NBs, filamentous F-actin was significantly enhanced after 0.5 and 2 h, and the fluorescence intensity and quantity of F-actin increased more significantly after 4 h, compared with the LIPUS group (Fig. 5A, B). Next, we evaluated the F/G-actin ratio according to the above time points. Compared with the control group, the ratio decreased at 0.5 h and 2 h, and increased significantly at 4 h in the LIPUS group. The LIPUS + cRGD-NBs group showed an increased ratio (especially at 4 h) compared with the LIPUS group (Fig. 5C, D). These results suggest that the microfilaments in the LIPUS group underwent a brief depolymerization process and then showed an increased polymerization. The actin polymerization was more pronounced after LIPUS was combined with cRGD-NBs.

### Reduced osteogenic ability of LIPUS/cRGD-NBs after TRPM7 inhibition

**Fig. 6:**
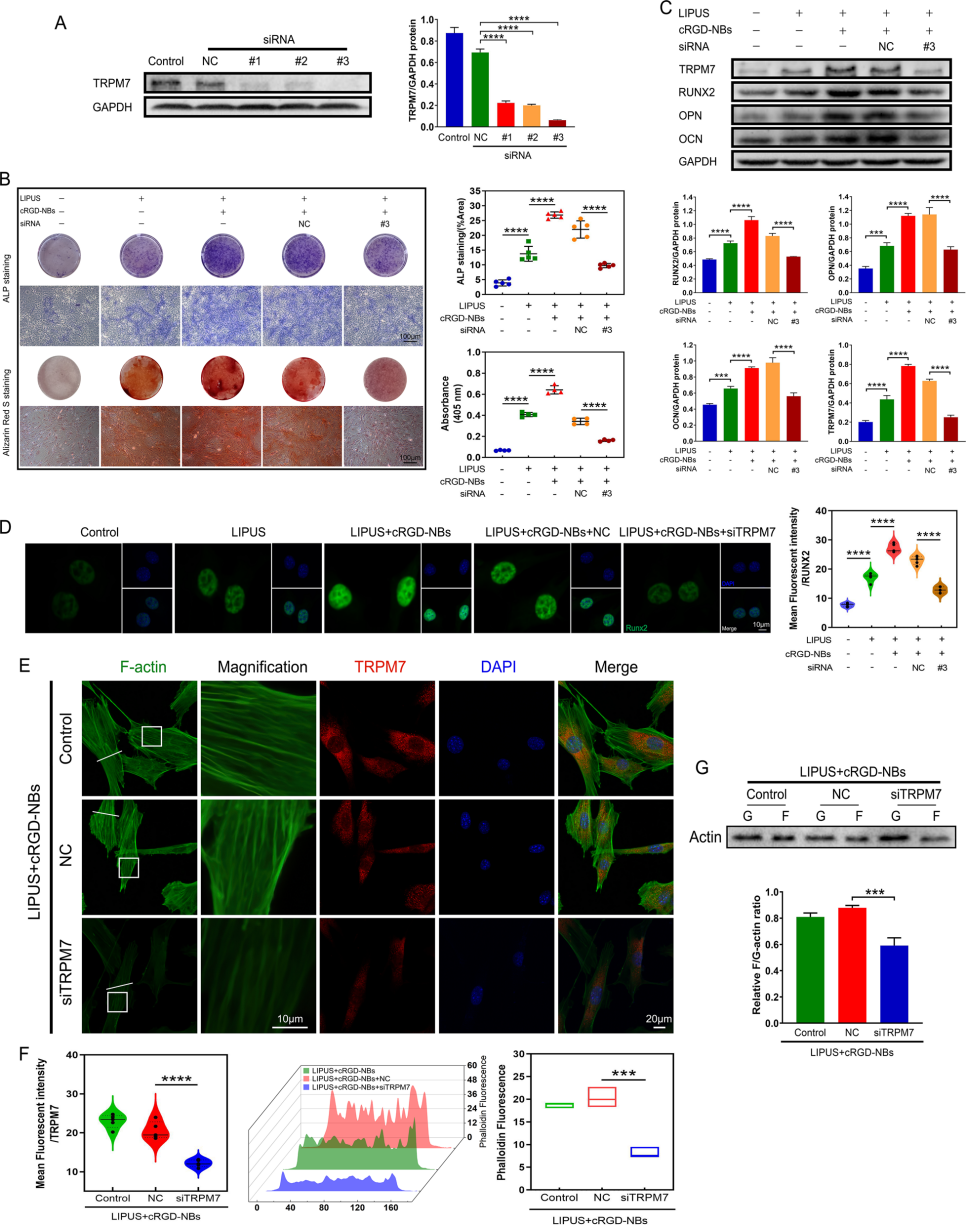
**A** The silencing efficiency of TRPM7 siRNA was assessed by WB and its quantitative analysis. **B** ALP staining and Alizarin Red S staining and related quantitative analysis were performed on day 7 and day 21, respectively.** C** The effect of each group on the level of osteogenic differentiation-related proteins (RUNX2, OPN, OCN, and TRPM7) after 14 days of treatment was assessed by WB and its quantitative analysis. **D** Immunofluorescence images stained with RUNX2 (green) and DAPI (blue) and related quantitative analysis. **E** Immunofluorescence images of TRPM7 (red), F-actin (green), and DAPI (blue) after LIPUS treatment for 4 h. **F** Quantification of TRPM7 fluorescence intensity in panel E and actin fluorescence intensity along the white lines in panel E. **G** F-actin and G-actin levels after LIPUS treatment for 4 h were assessed by WB and its quantitative analysis. Data are presented as mean ± SD (*n* = 3). ***P < 0.001, ****P < 0.0001

We used TRPM7 interference sequences to assess the effect of TRPM7 on the enhanced osteogenesis of LIPUS combined with cRGD-NBs. WB analysis showed that the protein levels of TRPM7 treated with three siRNA-TRPM7 sequences were significantly reduced compared with the siRNA-NC group. The siRNA#3 caused the lowest level of TRPM7 protein and was used for the subsequent experiments (Fig. 6A). ALP staining and Alizarin Red S staining showed a significant increase in both blue-purple staining and calcium nodule deposition in the LIPUS + cRGD-NBs group compared with the LIPUS group. Compared with the LIPUS + cRGD-NBs + NC group, the positive staining areas of the LIPUS + cRGD-NBs + siTRPM7 group were significantly reduced (Fig. 6B). WB analysis showed higher protein expression of RUNX2, OPN, OCN, and TRPM7 in LIPUS + cRGD-NBs group than LIPUS group. Moreover, these protein expression were significantly downregulated in the LIPUS + cRGD-NBs + siTRPM7 group compared with the LIPUS + cRGD-NBs + NC group (Fig. 6C). Immunofluorescence assay for RUNX2 expression revealed a significantly reduced fluorescence intensity in the LIPUS + cRGD-NBs + siTRPM7 group compared with the LIPUS + cRGD-NBs + NC group (Fig. 6D). These results indicate that TRPM7 could play an important role in the effect of cRGD-NBs on osteogenesis promoted LIPUS.

To clarify the possible relationship between TRPM7 and actin microfilaments in the process of BMSCs osteogenesis promoted by LIPUS/cRGD-NBs, the fluorescent phalloidin revealed F-actin dynamics of BMSCs treated by inhibition of TRPM7. Compared with the LIPUS + cRGD-NBs + NC group, the TRPM7 fluorescence intensity in the LIPUS + cRGD-NBs + siTRPM7 group was significantly decreased, accompanied by a significant decrease in the quantity and fluorescence intensity of F-actin (Fig. 6E, F). WB analysis showed that the F/G-actin ratio of the LIPUS + cRGD-NBs + siTRPM7 group was significantly lower than that of the LIPUS + cRGD-NBs + NC group (Fig. 6G). These results indicate that the inhibition of TPRM7 could reduce the polymerization of actin microfilaments.

### Possible regulatory role of actin polymerization in the osteogenesis promoted by LIPUS combined with cRGD-NBs

**Fig. 7:**
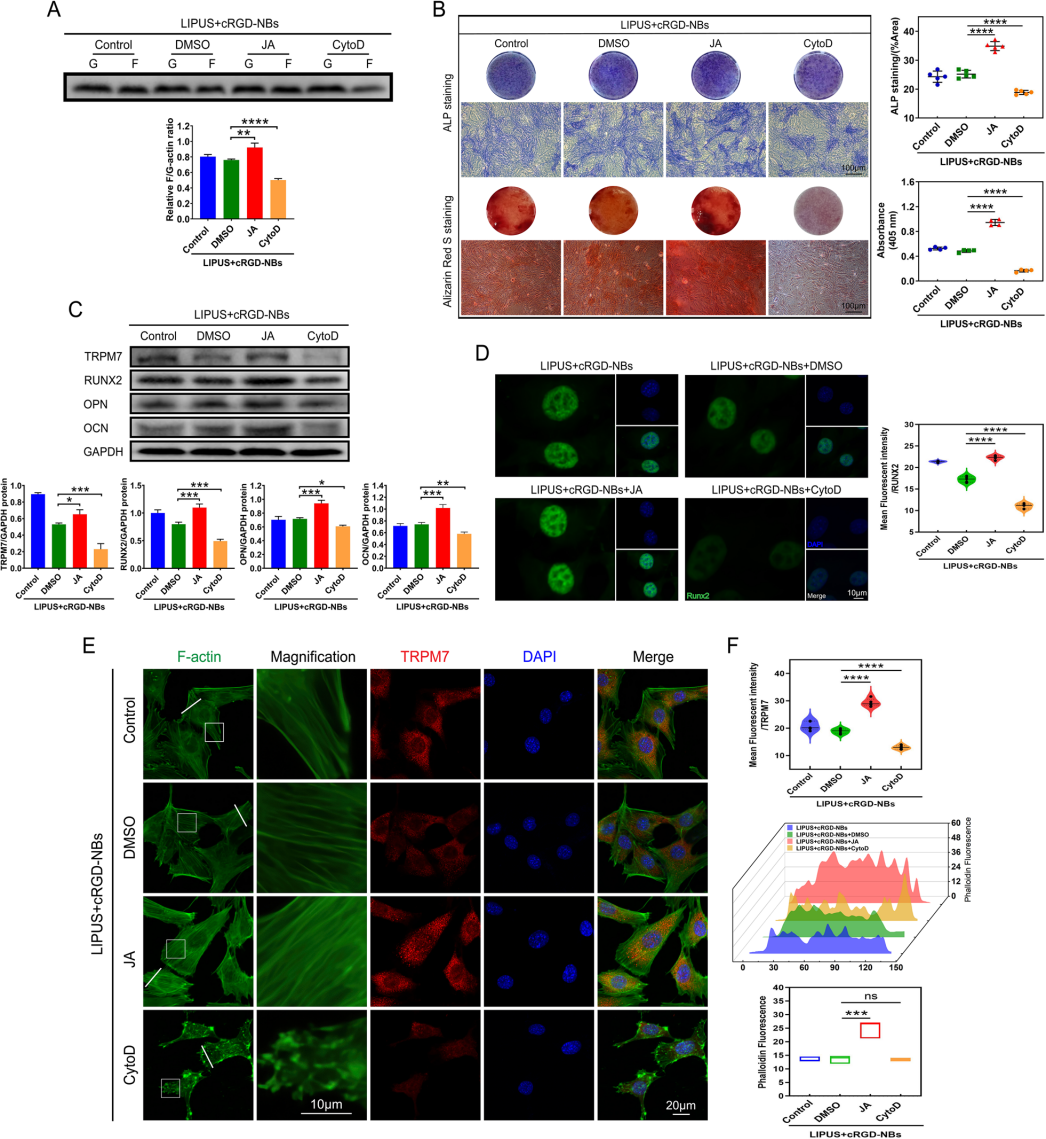
**A** F-actin and G-actin levels in each group were assessed by WB and its quantitative analysis. **B** ALP staining and Alizarin Red S staining and related quantitative analysis were performed on day 7 and day 21, respectively. **C** The effect of each group on the level of osteogenic differentiation-related proteins (RUNX2, OPN, OCN, and TRPM7) after 14 days of treatment was assessed by WB and its quantitative analysis. **D** Immunofluorescence images stained with RUNX2 (green) and DAPI (blue) and related quantitative analysis. **E** Immunofluorescence images stained with TRPM7 (red), F-actin (green), and DAPI (blue). **F** Quantification of TRPM7 fluorescence intensity in panel E and actin fluorescence intensity along the white lines in panel E. Data are presented as mean ± SD (*n* = 3). *P < 0.05, **P < 0.01, ***P < 0.001, ****P < 0.0001

To further examine the effect of actin polymerization on the enhanced osteogenesis ability of LIPUS combined with cRGD-NBs, BMSCs were treated with CytoD and JA, which can depolymerize and polymerize F-actin, respectively [35]. All groups were treated with the LIPUS + cRGD-NBs. Compared with the DMSO group, the F/G-actin ratio increased significantly in the JA group and decreased significantly in the CytoD group (Fig. 7A). Moreover, the positive staining areas in the JA or CytoD groups were significantly increased or reduced, respectively, compared with the DMSO group (Fig. 7B). WB analysis showed that, compared with the DMSO group, the RUNX2, OPN, OCN, and TRPM7 protein levels in the JA group were significantly increased, while those of the CytoD group were significantly reduced (Fig. 7C). Immunofluorescence assay for RUNX2 expression showed a similar trend of the WB analysis (Fig. 7D). These results indicate that the polymerization of actin played a key role in the osteogenesis of BMSCs promoted by LIPUS combined with cRGD-NBs.

After JA treatment, a significant increase was observed in the amount and fluorescence intensity of F-actin, accompanied by a significant increase in the fluorescence intensity of TRPM7, compared with the DMSO group. After CytoD treatment, the amounts of intracellular fluorescence dots were significantly increased, accompanied by the reduction in the fluorescence intensity of TRPM7, compared with the DMSO group (Fig. 7E, F). These results suggested that the polymerization of actin microfilaments could influence the expression of TPRM7.

### Possible role of TRPM7-mediated intracellular calcium oscillations in osteogenesis of BMSCs promoted by LIPUS/cRGD-NBs combination

**Fig. 8:**
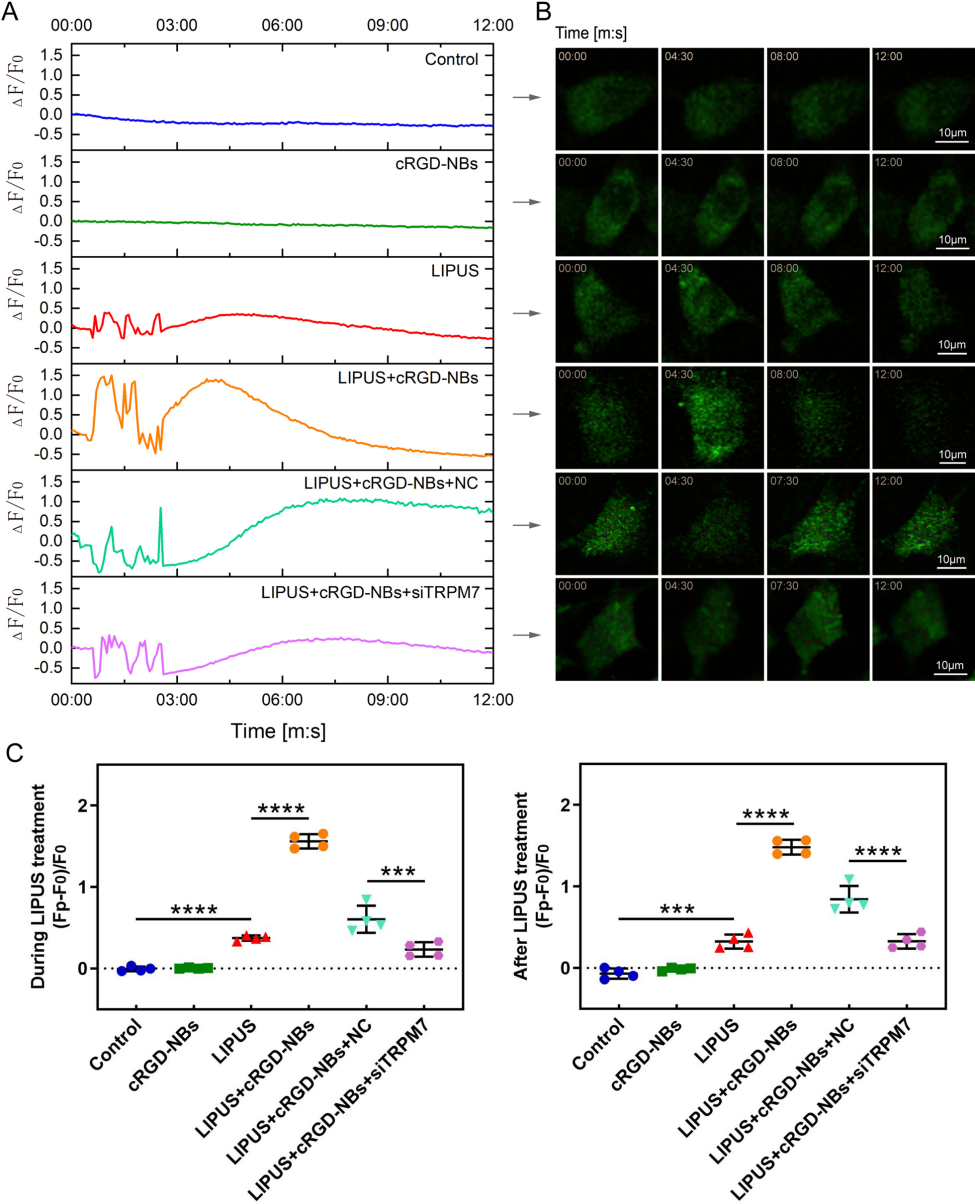
**A** Time profile of Ca^2+^ reaction on a single cell; the LIPUS treatment window was 00:30–2:30, and the total observation time was 12 min. **B** Fluorescence images of Ca^2+^ reaction taken at different time points, corresponding to panel A. **C** Comparison of highest *F*_P_ values of each group during LIPUS treatment (00:30–2:30) and after LIPUS treatment (2:30–12:00). Data are presented as mean ± SD (*n* = 4). ***P < 0.001, ****P < 0.0001

To further understand whether intracellular calcium signaling responds to mechanical stress under LIPUS/cRGD-NBs, the intracellular Ca^2+^ concentration was measured in real time via laser confocal microscopy with the fluorescent probe Fluo-4 AM. The intracellular Ca^2+^ concentration increased significantly in LIPUS group compared with that of the control group. After the application of cRGD-NBs, the peak Ca^2+^ fluorescence intensity induced by LIPUS was significantly increased compared with that measured with LIPUS alone (Fig. 8A–C). These results indicate that the combination of LIPUS with cRGD-NBs can further promote the increase in intracellular Ca^2+^ concentration. The peak Ca^2+^ fluorescence intensity during and after LIPUS treatment were significantly decreased in LIPUS + cRGD-NBs + siTRPM7 group, compared with that of the LIPUS + cRGD-NBs + NC group (Fig. 8A–C). These results illustrate that the extracellular Ca^2+^ influx, controlled partly by TRPM7, could participated in the effect of LIPUS combined with cRGD-NBs on BMSCs.

## Discussion

Many researches in animals and patients have highlighted the good performance of LIPUS in accelerating fresh fracture healing and treating nonunion fractures. However, the detailed mechanism is unclear [5, 36, 37]. Moreover, the efficacy of LIPUS needs to be further optimized because of the long treatment period and limited effect on deep bone fracture. Our study showed that LIPUS/cRGD-NBs could enhance the ability of LIPUS to promote osteogenesis of BMSCs in vitro and cranial bone defect in vivo. The interaction between the actin cytoskeleton, the mechanosensitive ion channel TRPM7, and the intracellular calcium signaling is important in regulating this process.

It has been shown that any biological effect of ultrasound that is accompanied by a temperature increment of less than 1 °C above the normal physiological level is referred to as a mechanical effect. Therefore, the biological effects of LIPUS in this study were mainly mediated by non-thermal mechanical effect [38]. LIPUS induced mechanical stress and strain can affect cytoskeleton rearrangement, which transmit forces from the extracellular matrix to the intracellular compartment [39]. The integrin receptors on the cell membrane are the major mechanosensory components, which connect to the actin cytoskeleton responsible for the transformation of mechanical stimuli into the biochemical reactions [40]. The cRGD is an active targeting peptide and one of the most important ligand of the integrin receptor [41]. In this study, cRGD-modified nanobubbles can actively target integrin receptors on BMSCs surface, which could rapidly transmit LIPUS mechanical effect into intracellular response. However, the non-targeting nanobubbles did not improve the ability of LIPUS to promote osteogenesis, which may be due to the small number of nanobubbles attached to the cell membrane and the lack of enough effective induction to enhance the effect of LIPUS.

It has been reported that the TRPM7 composed of an ion channel and an enzymatically active kinase domain mediates the Ca^2+^ influx through the plasma membrane. As a mechanosensitive channel, TRPM7 activity is detected at lipid rafts and controlled by the cell mechanical cues [42]. Under LIPUS treatment, cRGD-NBs undergo volume pulsations and generate further fluid microflows around them, which increase the local shear forces applied to the cells. cRGD-NBs binding to the cell membrane could act as local “nanomechanical force generators” to induce conformational changes in membrane proteins/receptors. LIPUS/cRGD-NBs could affect the TRPM7 activity by conformational changes associated with Ca^2+^ channel opening, which activated specific signaling pathways [43].

External mechanical stimuli can be delivered to the actin microfilaments and alter the structure of the cytoskeleton to activate biological effect [44–46]. Previous studies have shown that increased actin polymerization and formation of peripheral actin bundles is related to the osteogenesis of BMSCs [47–50]. Under electrical stimulation, the actin cytoskeleton of adipose stem cells rearranged into more aligned F-actin network, which promotes osteogenesis differentiation [51]. The depolymerized F-actin resulted in the inhibition of the osteogenesis of BMSCs in the microgravity [52]. It has been suggested that actin microfilaments polymerization play a role in osteogenesis induced by mechanical stress [53]. After the LIPUS treatment, the transient depolymerization could result in the reorganization of the actin microfilaments and then further promote the polymerization of the microfilaments. In this study, the mechanical effect was rapidly transmitted to the intracellular microfilaments through the mechanical cRGD-NB–integrin–actin linkage. cRGD-NBs accelerated the polymerization of microfilaments promoted by LIPUS and activated intracellular signaling pathways.

Dynamic physical forces induce rearrangement of F-actin struts, leading to increased expression of osteogenic genes. The osteogenic commitment of MSCs can enhanced by actin polymerization, which can bind Yes-associated protein (YAP) complex and prevent its nuclear localization [54]. Intranuclear F-actin could lead to nuclear export of YAP, which releases the inhibition of nuclear YAP to RUNX2 initiation of osteogenesis [55]. Previous studies also showed that TRPM7 is also important for bone formation [56]. TRPM7 is one of key mechanical sensors involved in the osteogenesis of MSCs induced by various mechanical stimuli including shear stress and pressure [19, 32]. We observed that cRGD-NBs enhanced the expression of TRPM7 in the cell membrane and cytoplasm and the expression of RUNX2 in nuclear induced by LIPUS, which is regulated by actin polymerization. We therefore propose that rearrangement of the actin microfilaments linked by cRGD-NB–integrin–cytoskeleton mediate the effects of LIPUS on the expression levels of TRPM7 and RUNX2.

Some signaling molecules activated by Ca^2+^ ions and repeated Ca^2+^ oscillations are involved in osteogenic differentiation of MSCs [57, 58]. Recently, it has been reported that Ca^2+^ oscillations in bone cells enhance bone formation, and the inhibition of calcium signaling significantly reduces the ability of bones to adapt to mechanical stress. In MSCs, Ca^2+^ is delivered to the cytoplasm through gated and receptor-operated channels in the plasma membrane. The changes in Ca^2+^-influx via stretch-sensitive ion channels are known to modulate mesenchymal osteogenic differentiation [59, 60]. The activation of TRPM7 channel, as one of stretch-activated channels, resulted in Ca^2+^ release into the cytoplasm induced by oscillatory shear stress and electrical fields [61, 62]. In this study, the increasing of intracellular Ca^2+^ concentration accompanied by LIPUS/cRGD-NBs induced osteogenesis was markedly decreased when TRPM7 was knocked down. These results suggested that TRPM7-mediated Ca^2+^ influx was involved in the process of osteogenic differentiation promoted by LIPUS/cRGD-NBs.

We also observed that the inhibition of TPRM7 reduced actin polymerization, indicating a possible mechanism that TRPM7 activity could regulate dynamics of actin microfilaments. In the process of BMSCs osteogenesis, TRPM7 could regulate the organization of actin microfilaments and F-actin-membrane interactions by Ca^2+^ influx and Ca^2+^-binding protein [63, 64]. The influx of Ca^2+^, which act as a second messenger to activate ERK1/2 phosphorylation and perinuclear F-actin polymerization, regulated the proliferation of mouse embryonic osteoblasts [65]. The cofilin-1 activity in a Ca^2+^-dependent manner linked reorganization of the actin cytoskeleton [66]. Therefore, TRPM7 mediated influx of Ca^2+^ could promote actin polymerization through elevated intracellular Ca^2+^ concentration under LIPUS/cRGD-NBs treatment.

Under the action of LIPUS, cRGD-NB act as a nanomechanical force generator on the plasma membrane and transmit the mechanical stimulation into BMSCs, promoting osteogenesis related signal transduction. cRGD-NBs sense the mechanical force of LIPUS and transmit to actin microfilaments quickly by integrin receptors. Actin microfilaments make a rearranged response to form more F-actin. The polymerization of actin affects BMSCs osteogenesis by inducing the osteogenic gene expression including RUNX2 and TRPM7. In addition, the mechanically sensitive TRPM7 also respond the conformational changes of plasma menbrane and activate calcium channel to mediate the extracellular Ca^2+^ influx. The increased intracellular Ca^2+^ concentration could enhance the expression of osteogenesis-related genes and promote actin polymerization. In summary, cRGD-NBs can enhance the responses of actin microfilaments and mechanically sensitive ion channel TRPM7 to LIPUS and play roles in promoting the osteogenic differentiation induced by LIPUS. The crosstalk of actin microfilaments and TRPM7 further strengthened this process.

## Conclusions

In this study, we designed and prepared nanobubbles (cRGD-NBs) able to target the integrin receptors on the cell membrane and possessing good biocompatibility. The targeted nanobubbles can be attached to the cell membrane in large quantities and act as nanomechanical force generators, exerting mechanical forces on the cell membrane and intracellular region after LIPUS treatment; this activates the mechanosensitive TRPM7 ion channel to mediate the influx of extracellular calcium. Meanwhile, the actin cytoskeleton can rapidly respond to the incoming mechanical force, leading to increased polymerization, and the combination of the two effects results in enhanced osteogenesis. Furthermore, TRPM7 and actin may regulate each other during this process. In short, under the action of LIPUS, cRGD-NBs can be used as nanomechanical force generators on the cell membrane to regulate intracellular signal transduction and optimize the osteogenic effect of LIPUS. This study provides a promising approach for optimizing the efficacy of LIPUS in promoting fracture healing, and a prospect about the application of LIPUS combined with targeted nanobubbles as an effective mechanobiological tool for regulating gene expression and stem cell differentiation.

## Supplementary Information

**Additional file 1: Figure S1.** Light microscopy images were taken at 0 h, 6 h, 24 h, and 48 h after the preparation of nanobubbles and microbubbles. **Figure S2.** The waveform output at different intensities of LIPUS (100 mW/cm^2^, 200 mW/cm^2^, 300 mW/cm^2^), 50% duty cycle. The ultrasound interval was about 20.9 ms. **Figure S3. A** Temperature change (ΔT) plots for the control, LIPUS, LIPUS + NBs, and LIPUS + cRGD-NBs groups treated with LIPUS with an intensity of 100 mW/cm^2^. **B** The corresponding infrared thermal images. **Figure S4.** **A** Temperature change (ΔT) plots for the control, LIPUS, LIPUS + NBs, and LIPUS + cRGD-NBs groups treated with LIPUS with an intensity of 200 mW/cm^2^. **B** The corresponding infrared thermal images. **Figure S5.** **A** Temperature change (ΔT) plots for the control, LIPUS, LIPUS + NBs, and LIPUS + cRGD-NBs groups treated with LIPUS with an intensity of 300 mW/cm^2^. **B** The corresponding infrared thermal images. **Figure S6.** ALP activity, an early marker of osteogenic differentiation, was measured on day 7 and day 14 following LIPUS and cRGD-NBs treatment. Data are presented as mean ± SD (*n* = 3). *P < 0.05, **P < 0.01, ***P < 0.001. **Figure S7.** Flow cytometric detection of cell proliferation in each group after 3 days of treatment and its quantitative analysis. **Figure S8.** The overall cranial micro-CT images corresponding to the groups in Fig. 3G.

## Data Availability

All data used to obtain the present results are available within the paper and the Supporting Information.
