# Supplementary material for: Low-intensity pulsed ultrasound/nanomechanical force generators enhance osteogenesis of BMSCs through microfilaments and TRPM7
Source: J Nanobiotechnology. 2022 Aug 13;20:378. doi: 10.1186/s12951-022-01587-3 (PMC9375242; doi:10.1186/s12951-022-01587-3)
Supplement: Supplementary file 1 — Additional file 1: Figure S1. Light microscopy images were taken at 0 h, 6 h, 24 h, and 48 h after the preparation of nanobubbles and microbubbles. Figure S2. The waveform output at different intensities of LIPUS (100 mW/cm2, 200 mW/cm2, 300 mW/cm2), 50% duty cycle. The ultrasound interval was about 20.9 ms. Figure S3. A Temperature change (ΔT) plots for the control, LIPUS, LIPUS + NBs, and LIPUS + cRGD-NBs groups treated with LIPUS with an intensity of 100 mW/cm2. B The corresponding infrared thermal images. Figure S4. A Temperature change (ΔT) plots for the control, LIPUS, LIPUS + NBs, and LIPUS + cRGD-NBs groups treated with LIPUS with an intensity of 200 mW/cm2. B The corresponding infrared thermal images. Figure S5. A Temperature change (ΔT) plots for the control, LIPUS, LIPUS + NBs, and LIPUS + cRGD-NBs groups treated with LIPUS with an intensity of 300 mW/cm2. B The corresponding infrared thermal images. Figure S6. ALP activity, an early marker of osteogenic differentiation, was measured on day 7 and day 14 following LIPUS and cRGD-NBs treatment. Data are presented as mean ± SD (n = 3). *P < 0.05, **P < 0.01, ***P < 0.001. Figure S7. Flow cytometric detection of cell proliferation in each group after 3 days of treatment and its quantitative analysis. Figure S8. The overall cranial micro-CT images corresponding to the groups in Fig. 3G. [file 12951_2022_1587_MOESM1_ESM.docx]

Low-intensity pulsed ultrasound/nanomechanical force generators enhance osteogenesis of BMSCs through microfilaments and TRPM7

Huan Yao^1,2^, Liang Zhang^2^, Shujin Yan^2^, Yiman He^2^, Hui Zhu^2^, Yasha Li^1^, Dong Wang^2^, Ke Yang^1*^

^1^Pediatric Research Institute, Children’s Hospital of Chongqing Medical University, National Clinical Research Center for Child Health and Disorders, Ministry of Education Key Laboratory of Child Development and Disorders, China International Science and Technology Cooperation base of Child development and Critical Disorders, Chongqing Engineering Research Center of Stem Cell Therapy, Chongqing 400014, China.

^2^Department of Ultrasound, The First Affiliated Hospital of Chongqing Medical University, Chongqing 400016, China.

^*^Correspondence: yangke@hospital.cqmu.edu.cn

**Supplementary methods**

**Evaluation of temperature changes induced by different intensities of LIPUS combined with cRGD-NBs**

After the cells were seeded in 24-well plates, 1 mL of culture medium was added to each well, followed by 60 μL of cRGD-NBs and NBs, respectively. PBS was added to the control and LIPUS groups. LIPUS was irradiated at 100 mW/cm^2^, 200 mW/cm^2^, and 300 mW/cm^2^ respectively, and the irradiation time was 10 min. Temperature changes and thermography images were recorded by an infrared thermography camera (Fotric 226, Shanghai, China).

**ALP activity assay**

The ALP activity of BMSCs was detected at days 7 and 14 using the ALP IB test kit (Wako, Osaka, Japan) according to the manufacturer’s instructions. The absorbance at 405 nm was measured with a Multiscan GO spectrometer (Thermo Fisher Scientific, Waltham, MA). Cellular total protein was detected with a bicinchoninic acid (BCA) protein assay kit (Beyotime) by measuring the absorbance at 595 nm. ALP activity values were normalized to the relative concentrations of total cellular protein.

**Cell proliferation assay**

The cells were treated for 3 days and then harvested by trypsinization (Beyotime). The cell proliferation assay was performed using a BeyoClick™ EdU Cell Proliferation Kit with Alexa Fluor 488 (Beyotime), according to the kit manual. After staining, the cells were analyzed by FACS.


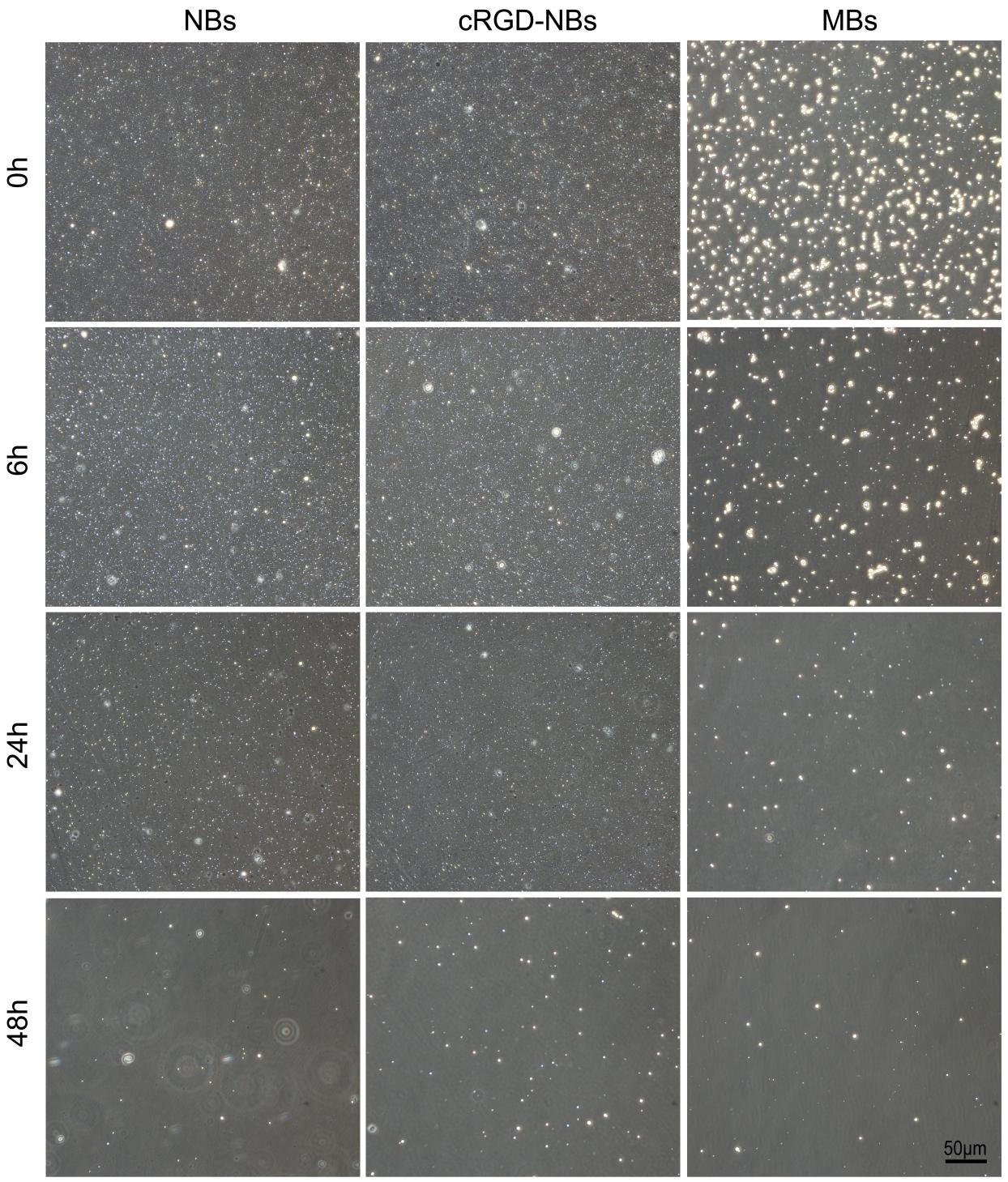


**Fig. S1** Light microscopy images were taken at 0 h, 6 h, 24 h, and 48 h after the preparation of nanobubbles and microbubbles.


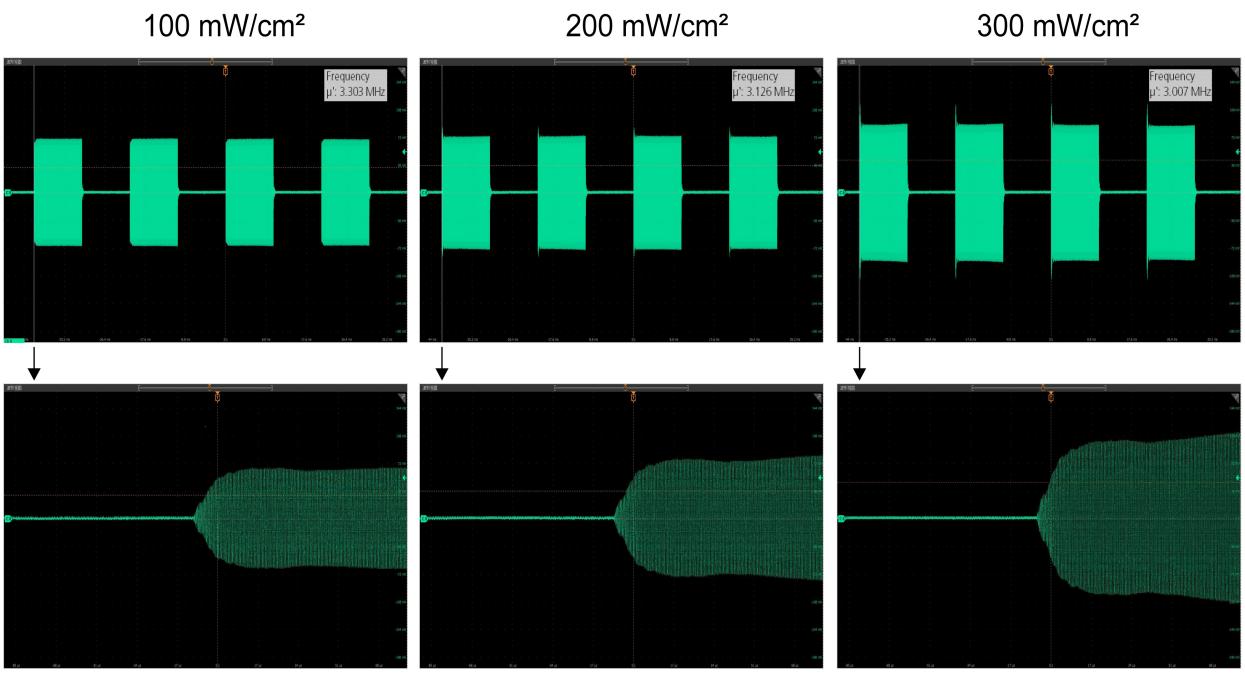


**Fig. S2** The waveform output at different intensities of LIPUS (100 mW/cm^2^, 200 mW/cm^2^, 300 mW/cm^2^), 50% duty cycle. The ultrasound interval was about 20.9 ms.


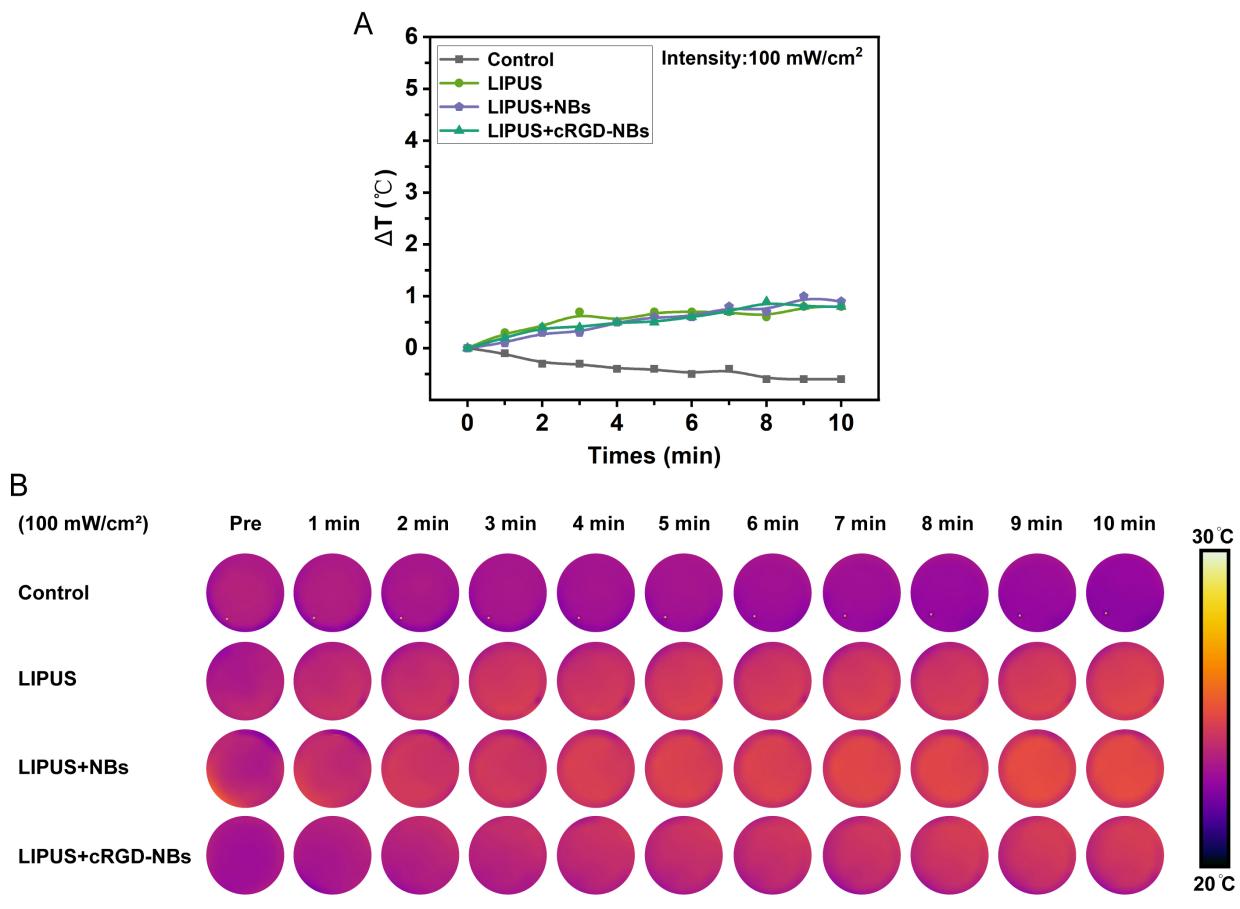


**Fig. S3 A** Temperature change (ΔT) plots for the control, LIPUS, LIPUS + NBs, and LIPUS + cRGD-NBs groups treated with LIPUS with an intensity of 100 mW/cm^2^. **B** The corresponding infrared thermal images.


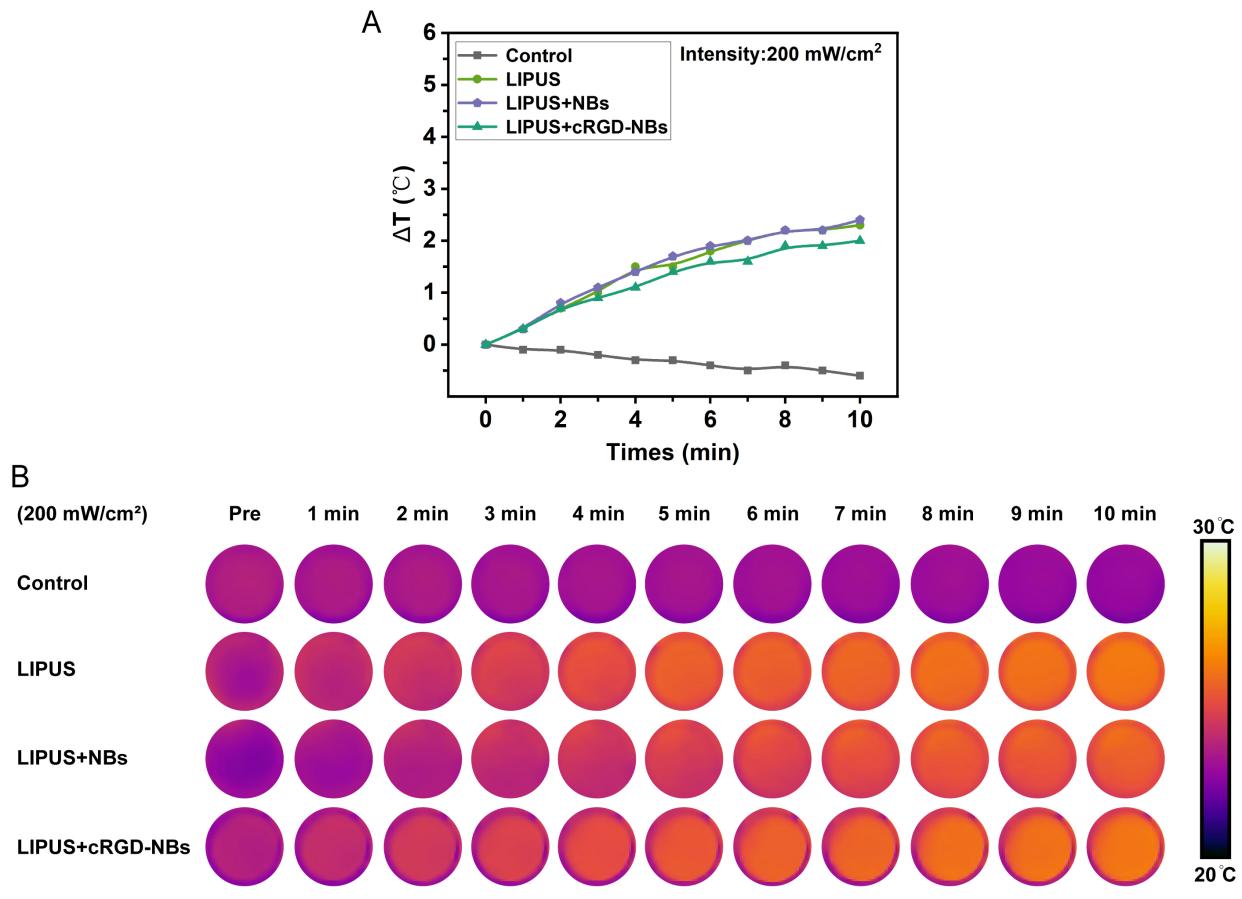


**Fig. S4 A** Temperature change (ΔT) plots for the control, LIPUS, LIPUS + NBs, and LIPUS + cRGD-NBs groups treated with LIPUS with an intensity of 200 mW/cm^2^. **B** The corresponding infrared thermal images.


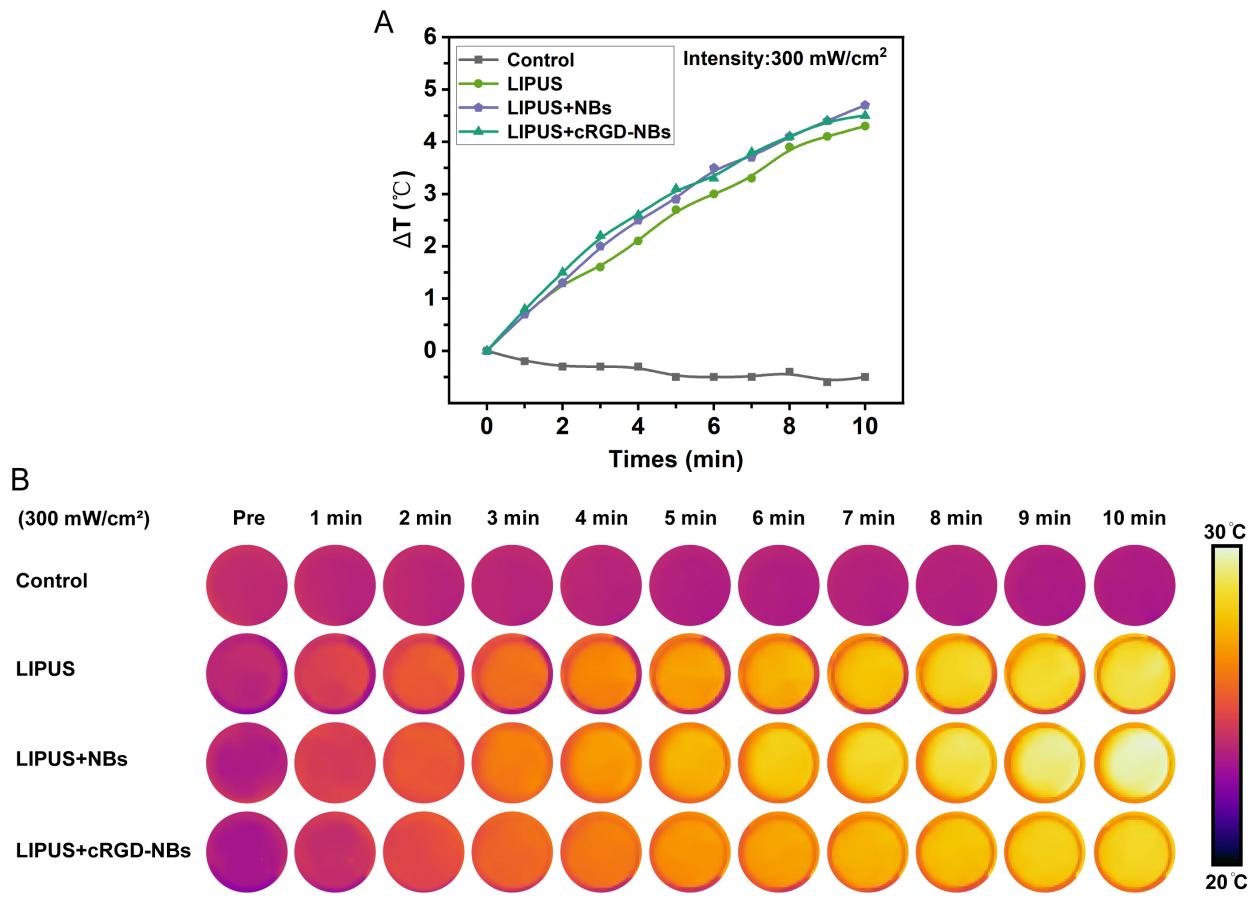


**Fig. S5 A** Temperature change (ΔT) plots for the control, LIPUS, LIPUS + NBs, and LIPUS + cRGD-NBs groups treated with LIPUS with an intensity of 300 mW/cm^2^. **B** The corresponding infrared thermal images.


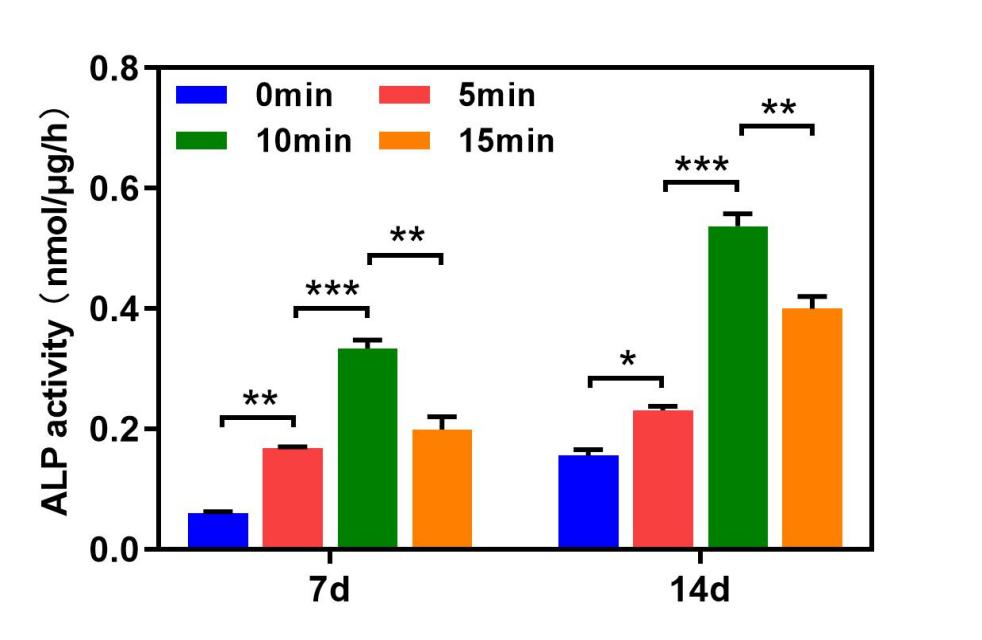


**Fig. S6** ALP activity, an early marker of osteogenic differentiation, was measured on day 7 and day 14 following LIPUS and cRGD-NBs treatment. Data are presented as mean ± SD (*n* = 3). * *P* < 0.05, ** *P* < 0.01, *** *P* < 0.001.

**
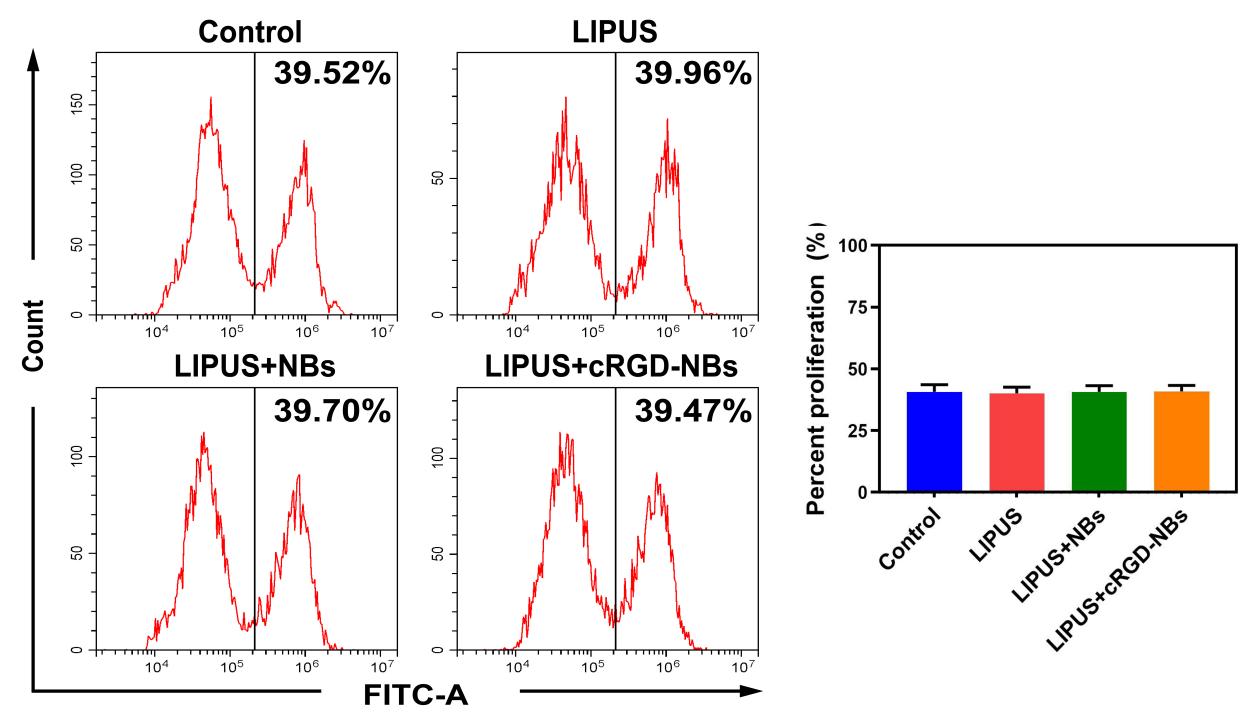
**

**Fig. S7** Flow cytometric detection of cell proliferation in each group after 3 days of treatment and its quantitative analysis.


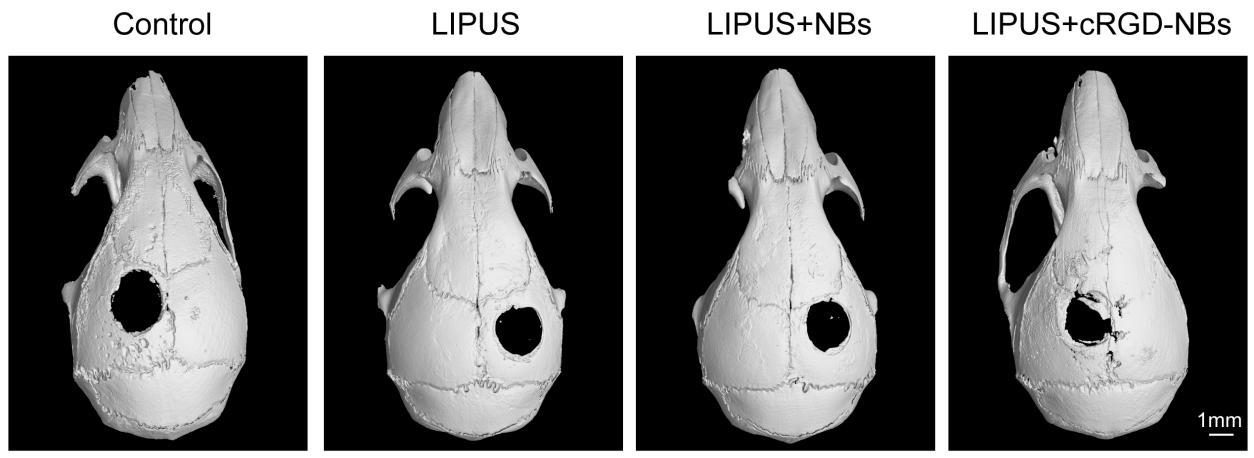


**Fig. S8** The overall cranial micro-CT images corresponding to the groups in Fig. 3G.
